# A five-collagen-based risk model in lung adenocarcinoma: prognostic significance and immune landscape

**DOI:** 10.3389/fonc.2023.1180723

**Published:** 2023-07-05

**Authors:** Lingjun Dong, Linhai Fu, Ting Zhu, Yuanlin Wu, Zhupeng Li, Jianyi Ding, Jiandong Zhang, Xiang Wang, Junjun Zhao, Guangmao Yu

**Affiliations:** Department of Thoracic Surgery, Shaoxing People’s Hospital, Shaoxing, Zhejiang, China

**Keywords:** LUAD, the extracellular matrix, collagen, tumor microenvironment, tumor-associated macrophages

## Abstract

As part of the tumor microenvironment (TME), collagen plays a significant role in cancer fibrosis formation. However, the collagen family expression profile and clinical features in lung adenocarcinoma (LUAD) are poorly understood. The objective of the present work was to investigate the expression pattern of genes from the collagen family in LUAD and to develop a predictive signature based on collagen family. The Cancer Genome Atlas (TCGA) samples were used as the training set, and five additional cohort samples obtained from the Gene Expression Omnibus (GEO) database were used as the validation set. A predictive model based on five collagen genes, including COL1A1, COL4A3, COL5A1, COL11A1, and COL22A1, was created by analyzing samples from the TCGA cohort using LASSO Cox analysis and univariate/multivariable Cox regression. Using Collagen-Risk scores, LUAD patients were then divided into high- and low-risk groups. KM survival analysis showed that collagen signature presented a robust prognostic power. GO and KEGG analyses confirmed that collagen signature was associated with extracellular matrix organization, ECM-receptor interaction, PI3K-Akts and AGE-RAGE signaling activation. High-risk patients exhibited a considerable activation of the p53 pathway and cell cycle, according to GSEA analysis. The Collage-Risk model showed unique features in immune cell infiltration and tumor-associated macrophage (TAM) polarization of the TME. Additionally, we deeply revealed the association of collagen signature with immune checkpoints (ICPs), tumor mutation burden (TMB), and tumor purity. We first constructed a reliable prognostic model based on TME principal component—collagen, which would enable clinicians to treat patients with LUAD more individually.

## 1 Introduction

Non-small cell lung cancer (NSCLC), which accounts for 80–85% of lung cancer occurrences worldwide, is the most common type of malignancy. 2,206,771 new cases of lung cancer were reported worldwide in 2020, making up 11.4% of all new cancer cases and placing lung cancer in second place globally (1, 2). There were 1796,144 new cases of lung cancer-related deaths, which is the highest number ever and accounts for 18% of all new cancer mortality cases (1, 2). The most common histological subtype of NSCLC, lung adenocarcinoma (LUAD), is followed by lung squamous cell carcinoma (LUSC), and LUAD accounts for more than 40% of NSCLC cases (3). Despite the use of cutting-edge technologies, strategies and therapies for lung cancer, only 17% of lung cancer patients survive for five years (4). Therefore, we urgently need to develop a precise way to forecast patients’ survival. The sensitivity of SCC, CEA and Cyfra 21-1, the most commonly used tumor marker, is only 20% to 50% (5), while other biomarkers, including ctDNA, EGFR, ALK and PD-L1, are fairly prevalent, they are not yet widely utilized in clinical practice (6). The advancement of sequencing technology has enabled bioinformatics analysis to pinpoint changes in gene expression, expanding the spectrum of potential methods for gauging the prognosis of LUAD patients. Therefore, in order to gain new insights into early illness diagnosis and the creation of customized therapy regimens, we set out to investigate the molecular signature that influences the onset and progression of LUAD.

The extracellular matrix (ECM) are scaffolds for tissues and organs (7). Many scholars wrongly believe that ECM is the “inanimate” or “static” portion of an organism. Our knowledge of the composition, structure, and function of the ECM has been considerably improved by recent fundamental research (8, 9). Collagen is the most ubiquitous protein in the human body and one of the most significant components of the ECM (10). There are currently 28 different varieties of the collagen family, and each collagen molecule has a basic structure that is either three homologous or heterologous trimer helix domain (11, 12). The amino acid GLY-X-Y repetitions, where X and Y are usually proline and 4-hydroxyproline, are the basic building block of triple-helical sequences. Depending on the changes in its structure and function, collagen can be categorized into a number of different types, including fibril-forming collagen, fibril-associated collagen, network-forming collagen, and membrane-anchored collagen (11–13).

Collagen protein serves as a scaffold of tumor microenvironment (TME). It impacts the TME, regulates ECM remodeling, and encourages tumor invasion, angiogenesis and migration by regulating collagen degradation and re-deposition (14). Tumor invasion and metastasis are caused by collagen loss in tumor tissue. For instance, in moderately differentiated colon cancer samples, collagen XV was nearly completely gone from the basement membrane. In human breast cancer, type XV and XIX collagen are gradually eliminated when ductal carcinoma *in situ* progresses to invasive carcinoma (15, 16). Collagen is an essential biomaterial for tumor angiogenesis. Numerous studies have shown that inhibiting collagen metabolism has an antiangiogenic effect and that the appropriate synthesis and deposition of collagen in the BM is required for vascular survival and development (14). However, the clinical characteristics and expression profile of the collagen family in LUAD are still unknown.

To address the thorny problems, we thoroughly examined the clinical characteristics and expression profile of the collagen family in LUAD. We thoroughly investigated the expression pattern and landscape of collagen family in LUAD to establish a Collagen-Risk model, which confirmed that can be used as an accurate and reliable biomarker. In addition, we obtained five GEO data sets as validation sets for the five collagen-based prognostic model for LUAD. Next, we performed functional analysis, immune infiltration analysis and immune check point analysis to provide new insights into the prognosis and immunotherapy of LUAD. Finally, a nomogram was developed to predict an individual overall survival (OS) based on the combination of the signatures of the five collagen genes and clinical features. In conclusion, our research may help with LUAD patient diagnosis and prognosis.

## 2 Materials and methods

### 2.1 Data sources

Standardized gene expression profiles of the TCGA-LUAD project were extracted from GDC data portal (https://portal.gdc.cancer.gov/)via perl language, including 598 lung tissue samples (59 normal cases and 529 LUAD cases). A total of 523 LUAD patients’ clinical observation data was available in TCGA database, which was served as a training set. Clinical observation information about the patients was gathered, including information on their survival time, survival status, age, gender, T pathological stage, N pathological stage, M pathological stage, and pathological stage. As validation sets, we downloaded five separate LUAD genomic profiles from the NCBI GEO database (https://www.ncbi.nlm.nih.gov/geo/): GSE13213, GSE31210, GSE72094, GSE30219, and GSE11969. GSE13213, GSE31210, GSE72094, GSE30219, and GSE11969 contain 117, 226, 442, 85, and 158 cases, respectively. **Table S1** shows the six cohorts’ basic clinical characteristics.

### 2.2 Identification of five collagens and generation of risk model

44 collagen family proteins were identified by the TCGA transcriptome and included in this research. 5542 differently expressed genes (DEGs) and 26 genes from the collagen family were found to be differentially expressed between normal and tumor tissues by using the ‘edge R’ package and setting the criteria to P< 0.01 and | log_2_(fold change) | > 1. Six collagen family proteins were discovered to be related to the prognosis of LUAD when the expression of collagen family proteins was examined using univariate Cox regression analysis to ascertain its correlation with OS in LUAD. Then, we used the ‘glmnet’ package to conduct a least absolute shrinkage and selection operator (LASSO) Cox regression model to integrate survival time, survival status, and six collagen genes’ expression data, and figured out five collagen family proteins (COL1A1, COL4A3, COL5A1, COL11A1, and COL22A1) which were considered to be crucial in LUAD. Through the modeling procedure outlined above, a five-collagens-based risk model was created, which entailed taking into consideration the expression of core genes as well as the associated risk coefficient. The model was defined in the equation: risk score = 0.036913204*COL1A1 + −0.094058955*COL4A3 + 0.005018018*COL5A1 + 0.011288384*COL11A1 + 0.08146509*COL22A1. Patients with LUAD were divided into high-risk and low-risk groups using the median risk score.

### 2.3 Survival analysis and the independent prognostic value of collagen-risk model

In this study, we used the ‘survival’ package, integrating gene expression data, patient survival time, patient survival status, and risk score, to evaluate the prognostic importance of each gene and risk model *via* the COX method. We initially conducted a univariate Cox Analysis of risk score to explore its association with prognosis in order to determine the independent prognostic validity of the collagen-risk model in LUAD and to investigate the relation of risk score with prognosis. All variables were then gathered to conduct a multivariable Cox analysis to assess whether risk score has independent prognostic value in predicting outcomes. ROC analysis was carried out to obtain AUC using the ‘pROC’ package. Specifically, we obtained patient follow-up time and collagen-risk score, and the ROC analysis was analyzed at 1-, 3-, 5-year time points, and the AUC and confidence interval were evaluated to obtain the final AUC values. The GSE13213, GSE31210, GSE72094, GSE30219, and GSE11969 datasets with OS and clinical information were used for external validation. The GSE31210 and GSE30219 datasets include recurrence-free survival (RFS) time, and we also analyzed the prognostic value of the collagen-risk model on RFS.

### 2.4 KEGG and GO enrichment analysis of collagen-risk model correlated genes

The GO annotation of the genes in the ‘org.Hs.eg.db’ package was used as the background set for the gene set functional enrichment study. The enrichment analysis was carried out using the ‘clusterProfiler’ package to acquire the findings of functional enrichment. The minimum gene set size was 5, the maximum gene set size was 5000, and statistical significance was defined as a P value of less than 0.05 and an FDR of less than 0.25.

### 2.5 GSEA analysis

We downloaded the Gene Set Enrichment Analysis (GSEA) software (version 3.0) from the GSEA website (http://software.broadinstitute.org/GSEA/index.jsp) and the “c2.cp.kegg.v7.4.symbols.gmt” subset from the Molecular Signatures Database to evaluate important pathways and molecular processes. The minimum and maximum gene sets were chosen at 5 and 5000, respectively, based on gene expression profiling and phenotypic grouping. 1000 resamples were used, and a P value of less than 0.05 and an FDR of less than 0.25 were deemed statistically significant.

### 2.6 Immune signature of collagen-risk model

In the present work, ‘Cibersort’ and ‘estimate’ package were used to estimate the abundance of immune cell infiltration, stromal score, immune score, and tumor purity for different risk groups. CIBERSORT is based on linear support vector regression to estimate the relative levels of 22 immune cells in different risk group, using standardized gene expression data to estimate immune cell infiltration (17). ESTIMATE is an algorithm for estimating the fraction of immune and stromal cells in tumor samples based on gene expression characteristics (18).

### 2.7 Tumor mutation status of collagen signature

Somatic mutation information of LUAD samples was downloaded from the Genomic Data Commons Data Portal (https://Portal.gdc.cancer.gov/). Tumor Mutation burden (TMB) is defined as the total number of mutations detected per million bases. By using the ‘maftool’ package, we determined the TMB for each LUAD sample and analyzed the significantly mutated genes across different risk groups, and also the interaction of mutated genes.

### 2.8 Generation collagen signature-based prognostic signature

We used the ‘rms’ package to integrate the data of patient survival time, patient survival status and seven clinical characteristics, including age, gender, tumor stage, T, M, N stage, and risk score of TCGA-LUAD samples, and established a nomogram by COX method (19). Meanwhile, a calibration chart comparing the predicted 1-, 3-, and 5-year survival probability and the actual situation was constructed to evaluate the consistency of the prognostic nomogram.

### 2.9 Sample collection

Lung samples were obtained from patients whose postoperative pathology was confirmed as LUAD and underwent cancer resection (Shaoxing People’s Hospital, Shaoxing, Zhejiang). Normal control tissue was obtained from lung tissue 5 cm beyond the edge of the cancerous tissue, and normal and cancerous tissues were eventually transferred to a -80 degree refrigerator for storage. All specimens were collected according to guidelines approved by the institutional review board at the Shaoxing People’s Hospital. The study complied with the ethical guidelines of the Declaration of Helsinki and was approved by the ethics committee of the Shaoxing People’s Hospital. Informed consent was obtained from each participant.

### 2.10 Quantitative real-time polymerase chain reaction

The mRNA expression levels of COL1A1, COL4A3, COL5A1, COL11A1, and COL22A1 were examined in lung cancer tissues and normal lung tissues. The primer sequences used for PCR are detailed in **Table S2**. Collected tissue specimens were homogenized with a homogenizer and total RNA was obtained from all tissues by using the SteadyPure Universal RNA Extraction Kit (Accurate Biology, AG21017, China) according to the manufacturer’s instructions. The Evo M-MLV Mix Kit with gDNA Clean for qPCR (Accurate Biology, AG11728, China) was used to reverse-transcribe 20 uL RNA into the cDNA. Relative expression of genes was quantified by the Universal SYBR Green Premix Pro Taq HS qPCR Kit (Accurate Biology, AG11701, China). The PCR amplification was carried out by the Applied Biosystems (USA). The 2^-ΔΔ^Ct method was used to calculate the relative expression levels.

### 2.11 Statistical analysis

All data were analyzed using R software. The Kaplan-Meier method and log-rank test were used to evaluate the OS between high- and the low-risk group. Nonparametric tests (log-rank test) were used to compare differences in ICPs, immune cell abundance, immune score, stromal score, tumor purity, and TMB between high- and low-risk groups. Continuous variables were analyzed by Wilcoxon test. Cox proportional hazards regression model was used to calculate the independent prognostic factors. P <0.05 was deemed statistically significant for all statistical analyses.

## 3 Results

### 3.1 Construction of collagen-risk model

**Figure 1:**
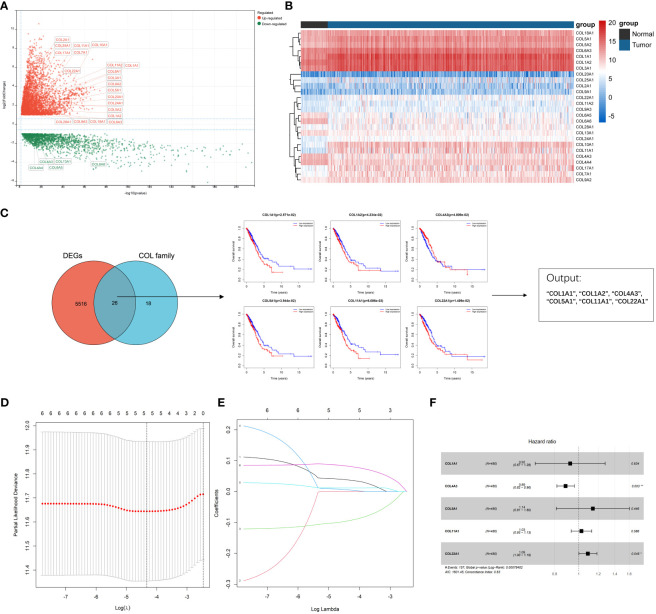
Identification of prognostic collagen family genes and construction of Collagen-Risk model in TCGA-LUAD. **(A)** The volcano plot presented the differentially expressed collagen family genes comparing normal tissues in LUAD. **(B)** Heatmap showed 26 DEGs of collagen family. **(C)** Univariate cox regression analysis for selecting alternative genes associated with OS of LUAD patients. The venn diagram showed the number of genes that cross between the DEGs and the collagen family genes. **(D)** LASSON coefficient profiles of prognostic collagen family genes. **(E)** Ten-fold cross-validated LASSON regression was used to identify five prognostic genes with minimum Lambda. **(F)** Forest plots for multivariate Cox regression analysis of five prognostic genes. *P < 0.05; **P < 0.01.

A total of 598 LUAD samples’ expression profile was downloaded from the TCGA database in order to identify collagen family proteins associated with prognosis and to find out the essential factors affecting the initiation and progress of LUAD. The criteria for this study was an FDR < 0.01, and | log_2_(fold change) | > 1. 44 collagen family proteins were enrolled in the TCGA-LUAD transcriptome data. According to the screening criteria, a total of 5542 genes, including 26 collagen family members, were significantly differently expressed in LUAD patients. The expression patterns and features of 26 DEGs in the collagen family are depicted in volcano plots and heat maps, with 5 collagen family genes downregulated and 21 collagen family genes upregulated, respectively (**Figures 1A, B**). Six of the 26 DEGs in the collagen family, according to univariate Cox regression analysis, were substantially associated with OS (**Figure 1C**). By using LASSO-Cox regression analysis, these genes were then further examined. The 5 most important genes, COL1A1, COL4A3, COL5A1, COL11A1, and COL22A1, were figured out through a lasso analysis (**Figures 1D, E**). COL4A3 and COL22A1 were shown to be independent prognostic risk variables according to the multivariate Cox analysis result (P < 0.05) (**Figure 1F**). Therefore, the final risk model was: Collagen-Risk score = 0.036913204*COL1A1−0.094058955*COL4A3 + 0.005018018*COL5A1 + 0.011288384*COL11A1 + 0.08146509*COL22A1.

### 3.2 The characteristic and prediction significance of the collagen-risk model

**Figure 2:**
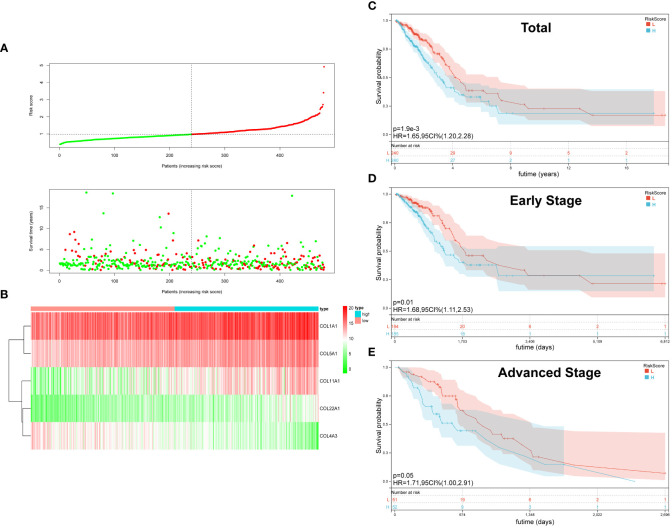
The Characteristic and Prediction Significance of the Collagen-Risk Model. **(A)** The distribution of Collagen-Risk score and survival status in TCGA-LUAD cohort. **(B)** Heatmap of the five identified collagen genes. **(C)** K-M survival plot showed the OS of total LUAD patients in high- and low-risk groups. **(D)** K-M survival plot showed the OS of early stage LUAD patients in high- and low-risk groups. Early-stage patients included stage I and II, n=369. **(E)** K-M survival plot showed the OS of advanced stage LUAD patients in high- and low-risk groups. Advanced stage patients included stage III and IV, n=103.

On the basis of the median value of the risk score values, all LUAD patients were categorized into high- and low-risk groups. Scatter plots were used to display the distribution of risk scores as well as the relationship between risk scores and survival status, and they revealed that the high-risk group experienced a higher death rate (**Figure 2A**). In LUAD, **Figure 2B** compares the expression of five genes from the collagen family in the high- and low-risk groups. While COL4A3 expression was higher in the low-risk group, that of COL5A1, COL1A1, COL1A2, and COL22A was upregulated in the high-risk group. OS was compared between high-risk and low-risk groups using the K-M survival analysis. According to the findings, the prognosis for patients in the high-risk group was worse (P=0.0019) (**Figure 2C**). In order to better explore the prognostic significance of Collagen-Risk score in different pathological stages of LUAD. We divided LUAD into early stage and advanced stage, with stage I and II as early stage and stage III and IV as advanced stage. In the early stages, we discovered that the Collagen-Risk score was a reliable indicator of OS, and that patients in the high-risk group had noticeably worse prognoses than those in the low-risk group. (p = 0.01) (**Figure 2D**). In another subgroup analysis, patients in the high-risk group had shorter OS than low-risk group in advanced stage, but the difference was not statistically significant (P=0.05) (**Figure 2E**).

### 3.3 Survival analysis of collagen-risk model

**Figure 3:**
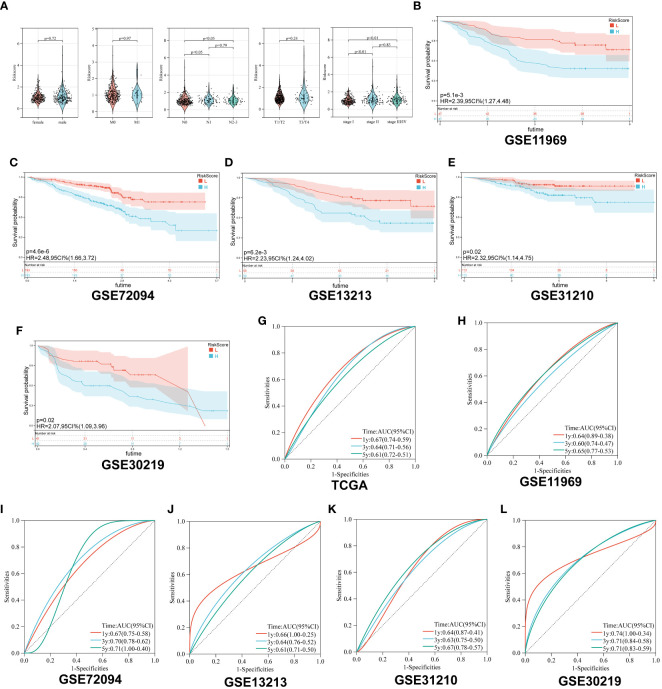
Survival analysis of Collagen-Risk model. **(A)** Difference analysis of distribution of Collagen-Risk scores in different gender, T, N, M, and tumor stage. Wilcoxon test was used to compare the statistical differences between the two groups, and Kruskal-Wallis test was used to compare the statistical differences among patients in three groups. **(B-F)** Validation of the prognostic value of Collagen-Risk model in five independent GEO datasets. **(G-L)** The time-dependent ROC curves of the Collagen-Risk signature in TCGA and GEO datasets.

First, we analyzed the distribution of collagen risk score in patients with different clinical characteristics, including gender, pathological stage, and TNM stage. We discovered that patients with higher N stage and pathological stage had higher risk score (N 1 vs N 0, p < 0.05; N 2-3 vs N 0, p < 0.05; Stage II vs Stage I, p < 0.01; Stage III/IV vs Stage I, p < 0.01). There was no significant difference in risk score between male and female patients, or between patients in M or T stages (P > 0.05) (**Figure 3A**).

Secondly, patients in the five GEO datasets were divided into high-risk and low-risk groups according to the median of Collagen-Risk score. We evaluated the performance of Collagen-Risk score in predicting OS using five GEO cohorts as external validation sets. As with the TCGA cohort survival analysis, patients in the low-risk group had longer survival in the validation datasets (P=0.0051, GSE11969; P<0.001, GSE72094; P=0.0062, GSE12313; P=0.02, GSE31210; P=0.02, GSE30219) (**Figures 3B-F**). RFS was significantly reduced in high-risk group than low-risk group, which indicated that Collagen-Risk score was an excellent predictor of RFS in LUAD patients (**Figures S1A, B**). Next, to further prove the superiority of this model in predicting the prognosis of lung cancer, ROC curve was used to assess ability of Collagen-Risk model, and AUC ranged from 0.60 to 0.74 in six data sets (**Figures 3G-L**). In general, the time dependent AUC indicated that in the TCGA and GEO datasets, the Collagen-Risk score had a considerable value in predicting OS in patients with LUAD (**Figure S1C**).

### 3.4 Diagnostic value of collagen-risk model in LUAD

**Figure 4:**
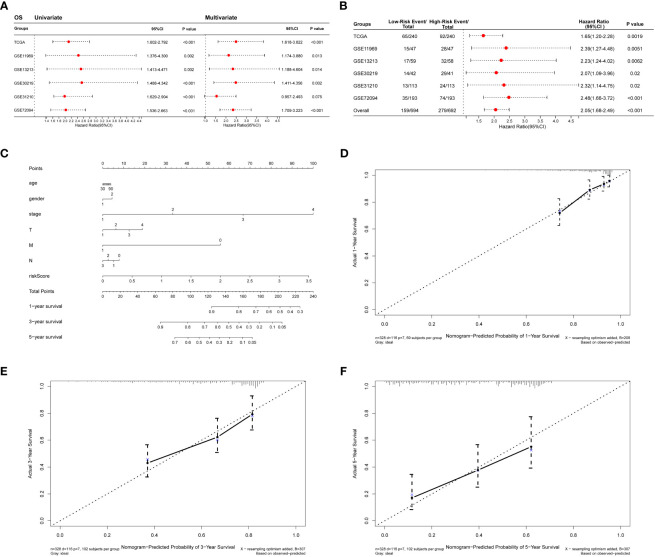
Diagnostic value of Collagen-Risk model in LUAD. **(A)** Forest plot of Cox analysis in TCGA and GEO datasets. For multivariate Cox regression analysis in TCGA dataset, HR value of Collagen-Risk model was adjusted by age, gender, TNM stage, and tumor stage. For multivariate Cox regression analysis in GSE11969 dataset, HR value of Collagen-Risk model was adjusted by age, gender, TN stage, and smoking history. For multivariate Cox regression analysis in GSE13213 dataset, HR value of Collagen-Risk model was adjusted by age, gender, TN stage, smoking history, EGFR status, and P53 status. For multivariate Cox regression analysis in GSE30219 dataset, HR value of Collagen-Risk model was adjusted by age, gender, and T stage. For multivariate Cox regression analysis in GSE31210 dataset, HR value of Collagen-Risk model was adjusted by age, gender, tumor stage, and smoking history. For multivariate Cox regression analysis in GSE72094 dataset, HR value of Collagen-Risk model was adjusted by age, gender, race, tumor stage, and smoking history. **(B)** A meta-analysis of verification result of five independent GEO datasets. **(C)** A nomogram based on age, gender, tumor stage, T, M, N stage and risk score. Gender, 1: Male, 2: Female. **(D)** Calibration curves showing the accuracy of nomogram predicting 1-year OS. **(E)** Calibration curves showing the accuracy of nomogram predicting 3-year OS. **(F)** Calibration curves showing the accuracy of nomogram predicting 5-year OS.

The collagen risk model’s predictive power for prognosis was investigated using univariate and multivariate Cox regression analyses. We evaluated the prognostic significance of Collagen-Risk model by univariate and multivariate Cox regression analyses in the TCGA and Geo datasets. The results of univariate Cox regression analysis showed that Collagen-Risk scores were significantly associated with OS in both TCGA LUAD cohort and the 5 GEO cohorts (TCGA, HR=2.115, 95%CI=1.602-2.792, P<0.001; GSE11969, HR=2.432, 95%CI=1.376-4.300, P=0.002; GSE13213, HR=2.514, 95%CI=1.413-4.471, P=0.002; GSE30219, HR=2.541, 95%CI=1.488-4.342, P<0.001; GSE31210, HR=2.174, 95%CI=1.6922.901, P<0.001; GSE372094, HR=2.022, 95%CI=1.536-2.663, P<0.001) (**Figure 4A**). Multivariate Cox regression analysis proved the Collagen-Risk scores to be a significant predictor for OS in both TCGA LUAD cohort and the four GEO cohorts (P<0.001, HR=2.49, 95%CI=1.618-3.822, TCGA; P=0.013, HR=2.134, 95%CI=1.174-3.880, GSE11969; P=0.014, HR=2.339, 95%CI=1.188-4.604, GSE13213; P=0.002, HR=2.479, 95%CI=1.411-4.356, GSE30219; P<0.001, HR=2.347, 95%CI=1.709-3.223, GSE72094) (**Figure 4A**). Meanwhile, we analyzed the prognostic value of clinical characteristics included in different datasets through univariate and multivariate Cox regression analysis. In the testing set of the TCGA dataset, age, N stage, and risk score were found to be independent factor of prognosis. In the validation dataset, age and risk score were consistently identified as independent predictors of prognosis in both univariate and multivariate analyses. (**Figures S2A, B**). In addition, univariate and multivariate Cox regression analysis demonstrated that Collagen-Risk scores were an independent prognostic factor for RFS (**Figure S1D**). A meta-analysis was conducted to determine the association and prognostic significance of the Collagen-Risk score with the OS and RFS of LUAD patients by analyzing the prognostic outcomes of the TCGA and 5 GEO cohorts. It was confirmed by overall HR, that Collagen-Risk score was a risk factor for OS in LUAD patients (overall HR = 2.05, 95% CI = 1.68-2.49, P < 0.001) (**Figure 4B**). Likewise, Collagen-Risk score was a risk factor that affects RFS in two GEO cohorts (overall HR = 3.10, 95% CI = 1.96-4.91, P < 0.001) (**Figure S1F**).

The results of univariate and multivariate Cox analyses revealed that Collagen-Risk score was strongly associated with OS and RFS of LUAD patients. Then, a nomogram, combining Collagen-Risk score with clinical characteristics such as age, gender, T stage, N stage, M stage, and tumor stage, was constructed to predict the 1-, 3-, and 5-year survival probabilities of patients (**Figure 4C**). The nomogram’s calibration curves showed that the predicted survival rates are closely related to the actual survival rates at 1, 3, and 5 years (**Figures 4D-F**).

### 3.5 Biological mechanism related to collagen-risk model

**Figure 5:**
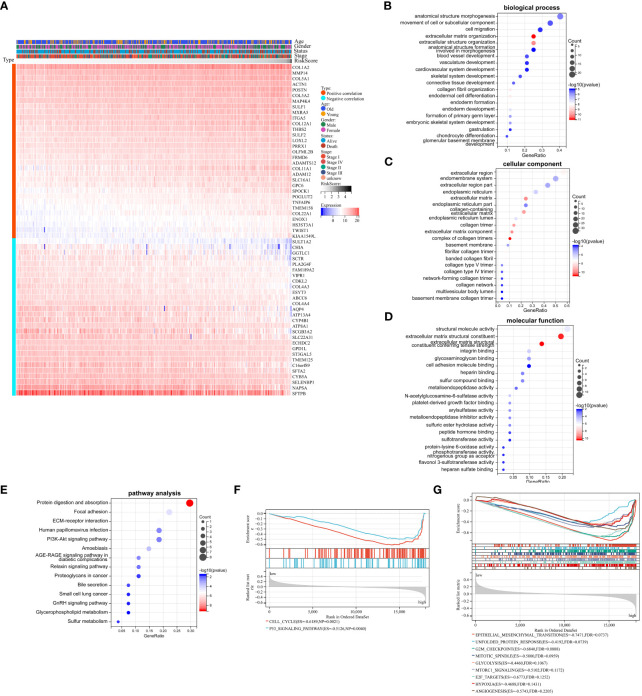
Biological mechanism related to Collagen-Risk model. **(A)** Heatmap of 59 Collagen-Risk model-associated genes in LUAD (Pearson | R| > 0.5, p < 0.05). GO **(B–D)** and KEGG **(E)** analysis of Collagen-Risk model correlated genes. **(F)** The KEGG pathway enrichment analysis based on GSEA. **(G)** The hallmark analysis based on GSEA.

We had demonstrated that Collagen-Risk model was an important prognostic factor in LUAD patients, and we would further investigate the mechanisms of its impact on prognosis. A Pearson correlation analysis was used to screen out collagen signature related genes (Pearson | R| > 0.5, p < 0.05). The results indicated that 59 genes were significantly associated with the Collagen-Risk model, with 31 genes having a positive correlation and 28 genes having a negative correlation (**Figure 5A**). These collagen signature correlated genes were then put to the teste for enrichment of GO and KEGG pathway. GO- biological process (BP) analysis of these genes showed enrichment mainly in the extracellular matrix organization, skeletal and cardiovascular system development, cardiovascular system development (**Figure 5B**, **Table S3**). Changes in cell composition (CC) were mainly concentrated in collagen trimer, extracellular region and extracellular matrix (**Figure 5C**, **Table S3**). Changes in molecular function (MF) were focused on protein binding, receptor and protease activity, and extracellular matrix structure (**Figure 5D**, **Table S3**). The analysis of KEGG signaling pathway showed that the genes were mainly involved in ECM-receptor interaction, cancer pathway, PI3K-Akts and AGE-RAGE signaling (**Figure 5E**, **Table S4**). Transcriptome data from TCGA-LUAD patients were used to identified Collagen-Risk score-associated signal transduction pathways by GSEA analysis. The KEGG analysis showed that CELL_CYCLE and p53_SIGNALING_PATHWAY was enriched in high-risk group (**Figure 5F**, **Table S5**). The hallmark analysis indicated that EPITHELIAL_MESENCHYMAL_TRANSITION, UNFOLDED_PROTEIN_RESPONSE, G2M_CHECKPOINT, MITOTIC_SPINDLE, GLYCOLYSIS, MTORC1_SIGNALING, E2F_TARGETS, HYPOXIA, and ANGIOGENESIS were mainly enriched in the high-risk group (**Figure 5G**, **Table S6**).

### 3.6 The relationship between collagen signature and immune signature

**Figure 6:**
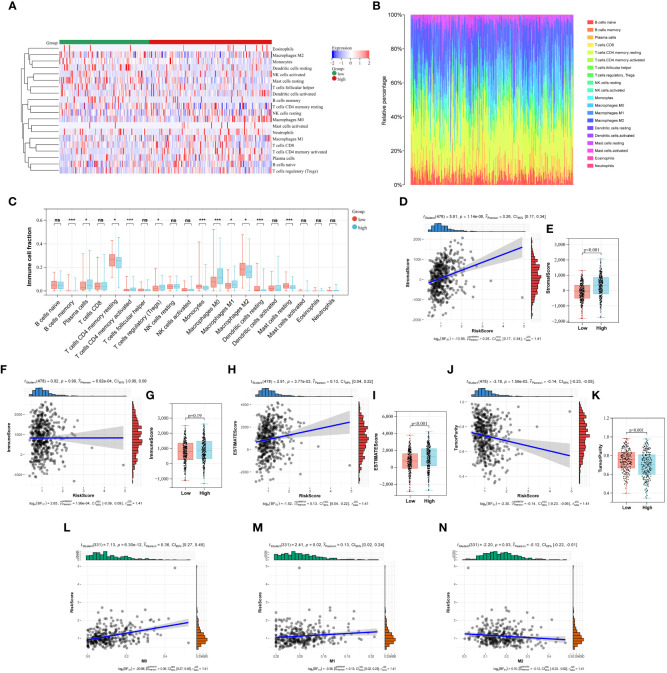
The relationship between Collagen-Risk score and immune signature. **(A)** Immune and stromal cell infiltration patterns in the low- and high-risk patients. **(B)** The proportions of 20 immune cells in LUAD patients. **(C)** Differences in immune cell infiltration between high- and low-risk groups. Analysis of differences between the two groups was performed by the Wilcoxon test. *P < 0.05; ***P < 0.001; ns not significant. **(D, F, H, J)** Association between stromal score, immune score, estimate score, tumor purity, and Collagen-Risk score. **(E, G, I, K)** Comparison of stromal score, immune score, estimate score, and tumor purity in high- and low-risk group. **(L-N)** The Collagen-Risk score and the infiltration levels of macrophage are estimated by Cibersort.

In this study, the abundance of 22 infiltrating immune cells in LUAD patients was calculated using CIBERSORT algorithm, and the content of T cells CD4 naive and T cells Gamma Delta was 0 in all samples. Meanwhile, P < 0.05 from the CIBERSORT results indicated that the immune infiltration analysis of the samples was credible, and 333 LUAD samples were left after eliminating the unqualified (**Figures 6A, B**). As shown in **Figure 6C**, the B cells memory, plasma cell, T cells CD4 memory resting, T cells CD4 memory activated, T cells regulatory (Tregs), Monocytes, Macrophages (M0), Macrophages (M1), Macrophages (M2), Dendritic cells resting and Mast cells resting were significantly different between low- and high-risk group (**Figure 6C**). Furthermore, we also used the ESTIMATE algorithm to calculate the immune score, stromal score, estimate score, and tumor purity for each sample. We found a positive correlation between stromal score, estimate score and Collagen-Risk score with Pearson coefficients of 0.26 and 0.13, respectively. However, tumor purity and Collagen-Risk score were negative correlated, and Pearson coefficients was -0.14 (**Figures 6D, F, H, J**). Immune score and stromal score were higher in high-risk group, while tumor purity was lower (**Figures 6E, G, I, K**).

The notion of the TME is new to the field of cancer research (20). ECM, fibroblasts linked to malignancy, vascular epithelial cells, and immune cells that have infiltrated make up the classic TME. Polarized macrophages, including the M1 and M2 subtypes, are among the several infiltrating immune cells that have been revealed in recent years to play important roles in tumor proliferation, invasion, and metastasis (21–23). Macrophages are incredibly pliable and form subcohorte in a particular TME, which is primarily divided into M1 and M2 types, with particular molecular and functional properties. M1 is essential for encouraging tumor growth, invasion, metastasis, and creating a suppressive immune milieu because it kills tumor cells and thwarts pathogen penetration. M2 has a direct impact on the survival, growth, stemness, invasion, angiogenesis, and immunosuppression of tumor cells (23, 24). We investigated in depth the correlation between collage signature and TAM infiltration. The levels of macrophage permeability between the high-risk and low-risk groups show a clear difference in **Figure 6C**. Next, we discovered a connection between TAM infiltration in the TCGA cohort and Collagen-Risk score. M0 and M1 macrophage infiltration was positively connected with the risk score, while M2 macrophage infiltration was negatively correlated (**Figures 6L, M**). The biomarkers of M1 included IL1A, Il1b, IL6, NOS2, TLR2, TLR4, CD80 and CD86. The biomarkers of M2 included CSF1R, MRC1, PPARG, ARG1, CD163, CLEC10A, RETNLB, PDCD1LG2 and CLEC7A (22). The link between the collagen-risk score and the M1 and M2 biomarkers was then examined. The M1 markers–IL1A, IL6, and NOS, as well as the M2 markers–RETNLB and PDCD1LG2, were all positively connected with the collagen-risk score, and TLR2 was negatively correlated with the Collagen-Risk score (**Figure S3**). Therefore, collagen signature may have predictive value for macrophage infiltration and typing.

Cancer-associated fibroblasts (CAFs) are the main components of the tumor stroma. CAFs act on tumor cells in multiple ways, such as aberrantly secreting ECM or remodeling ECM, secreting cytokines to cause metabolic reprogramming, promoting angiogenesis, and so on (25). Recent research has found that the effects of CAFs on tumor cells are diverse. Biomarkers (FAP, POSTN, PDGFRα/β, FSP-1, CD90, Palladin, OPN, AEBP1, TNC, CD10, and GPR77) represent cancer-promoting CAFs (26). These cells can promote tumor development by metabolic effects, thus promoting angiogenesis and immune suppression. However, Meflin+ CAF and CD146+ CAF are closely associated with better pathological histological features and prognosis of patients. Therefore, we investigated the relationship between these biomarkers and the collagen risk signature. The results of correlation analysis showed that risk score was positively correlated with both cancer-promoting and cancer-restraining CAFs biomarkers, suggesting that the activity of CAFs in high-risk patients was higher than that in low-risk patients, further confirming the importance of CAFs in tumor progression (**Figure S4**). However, further basic research is needed to explain the mechanisms involved in this relationship.

### 3.7 The relationship between collagen signature and immunotherapy-related biomarkers and TMB

**Figure 7:**
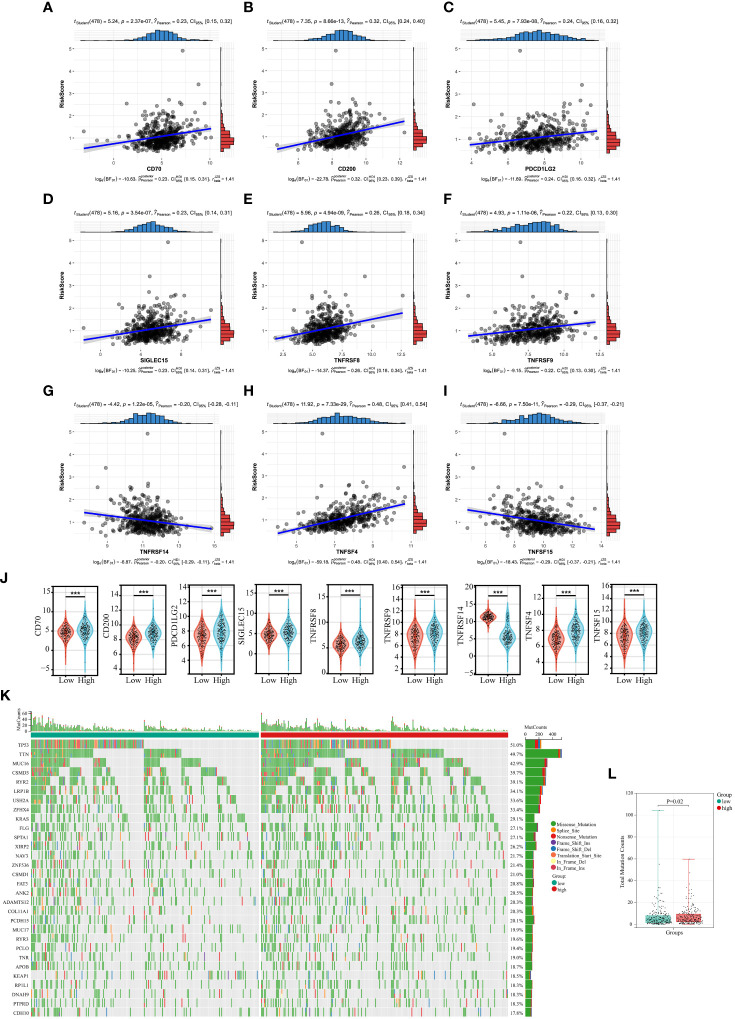
The relationship between Collagen-Risk score and immunotherapy-related biomarkers and TMB. **(A-I)** Association between CD70, CD200, PDCD1LG2, SIGLEC15, TNFRSF8, TNFRSF9, TNFSF4, and Collagen-Risk score. **(J)** Comparison of identified immune checkpoints in high- and low-risk group. ***P < 0.001. **(K)** The mutation profile of top30 genes in low- and high-risk groups. **(L)** Comparison of total mutation counts in the low- and the high-risk groups.

In recent years, tumor immunotherapy has been a hot spot in the field of cancer research and treatment, and has shown some promising and encouraging achievements (27, 28). The preliminary results of several clinical studies on immunotherapy for NSCLC suggested that immunotherapy could bring better survival benefit for patients with NSCLC (29–31). Subsequently, we investigated the correlation between expression of 35 immune checkpoints and Collagen-Risk score (32). The results suggested that the Collagen-Risk score had a strong positive correlation with CD70, CD200, PDCD1LG2, SIGLEC15, TNFRSF8, TNFRSF9, and TNFSF4, and had a strong negative correlation with TNFRSF14 and TNFSF15 (**Figures 7A-I**). Wilcoxon test confirmed that above 9 immune checkpoints were significantly different between low- and high-risk group (**Figure 7J**).

Each LUAD patient’s mutation profile was examined. Top 30 most significantly altered genes throughout the complete genome included TP53, TTN, MUC16, CSMD3, RYR2, LRP1B, USH2A, ZFHX4, KRAS, FLG, SPTA1, XIRP2, NAV3, ZNF536, CSMD1, FAT3, ANK2, DAMTS12, COL11A1, PCDH15, MUC17, RYR3, PCLO, TNR, APOB, KEAP1, RR1L1, DNAH9, PTPRD, CDH10 (**Figure 7K**). After that, we calculated the TMB for each sample and discovered that the high-risk score group had a considerably higher TMB (**Figure 7L**).

### 3.8 Experimental data validation of prognostic genes

**Figure 8:**
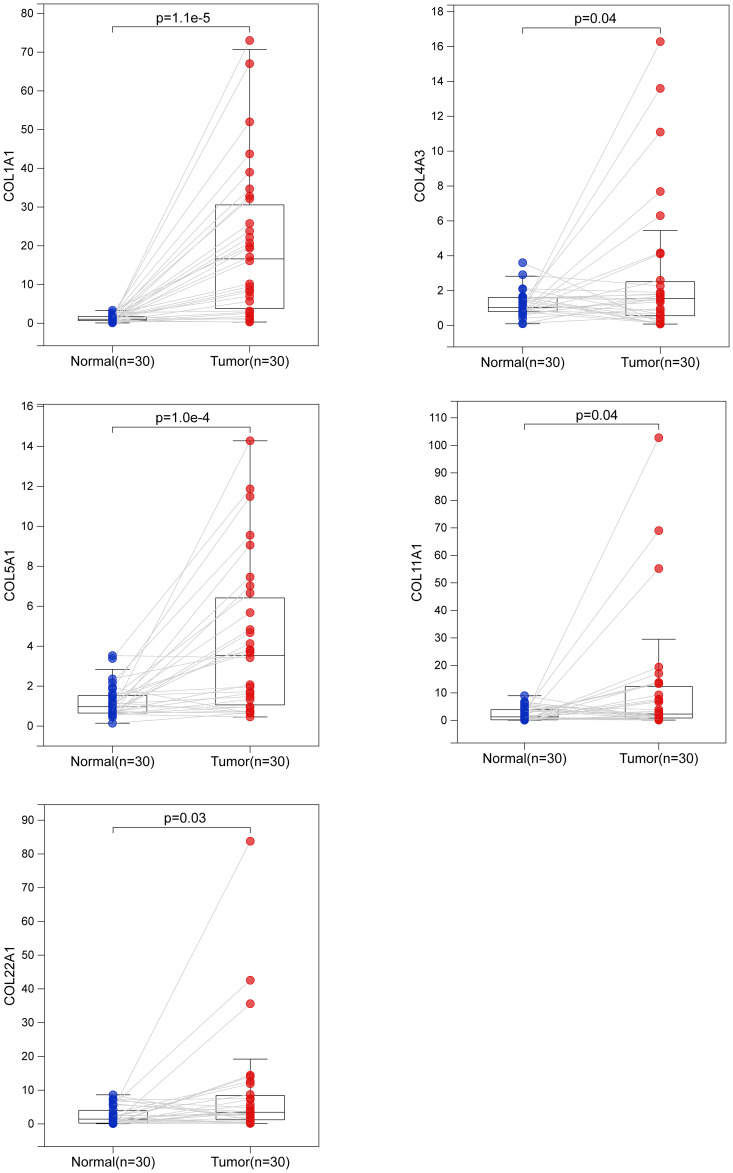
Biological verification of the expression of 5 collagen family genes in LUAD by quantitative real-time polymerase chain reaction. The difference of COL1A1, COL4A3, COL5A1, COL11A1 and COL22A1 mRNA expression between normal and LUAD tissues.

To verify these results, we examined the transcript levels of five collagen family proteins in human LUAD specimens. We collected a total of 30 cases each of normal and LUAD tissues. The results showed that the expression of the five collagen family proteins was much higher in most tumors than in normal tissues (**Figure 8**).

## 4 Discussion

One of the most prevalent malignant tumors, LUAD seriously affects people’s health worldwide (33). The rapid development of high-throughput sequencing technology has helped us discover more and more prognostic markers and immunotherapy targets (34, 35). Even though we have made considerable efforts to improve the treatment of LUAD, the mortality rate is still very high (36). Over the past few decades, scholars have come to realize the importance of ECM. In addition, ECM has become a biomarker and therapeutic target for cancer prediction, diagnosis and prognosis (37). However, there are still no biomarkers associated with ECM that accurately reflect the TME and prognosis of LUAD. Therefore, we firstly propose a risk model based on t collagen signature to predict the prognosis of LUAD. This study investigated the TCGA-LUAD data set and five GEO data sets to establish and validate the accuracy and specificity of Collagen-Risk model. In addition, Collagen-Risk score was associated with a number of important clinical features. Cox univariate and multivariate analyses demonstrated that Collagen-Risk score was an independent risk factor for the prognosis of LUAD. Subsequently, based on the TCGA data set, we found that immune cell subtypes, immune infiltration levels, TAM and ICP were strongly associated with the five-collagen-based signature. Through signal pathway analysis, we found that the strong prognostic ability of this Collagen-Risk model was attributed to unique extracellular matrix organization, protein binding, ECM-receptor interaction, cancer pathway, PI3K-Akts and AGE-RAGE signaling in the different risk groups. Finally, we integrated the collagen signature and clinical parameters to build a nomogram. The calibration curves demonstrate that at 3 and 5 years, the actual OS and nomogram-predicted OS were closely matched.

The fact that Collagen signatures exhibit strong predictive power may be because these members themselves (COL1A1, COL4A3, COL5A1, COL11A1, COL22A1) have been reported to predict tumor prognosis. COL1A1 has been reported to be closely associated with the prognosis of patients with lung adenocarcinoma, lung squamous carcinoma and esophageal cancer (38–40). Additionally, COL11A1 was shown to be significantly expressed in both NSCLC and lung adenocarcinoma, and it has been shown to encourage the growth, migration, and invasion of NSCLC cell lines *in vitro (* 41–43). The amount of plasma COL11A1 can aid with lung cancer diagnosis and prognosis (43). Researchers showed that COL4A3 was a significant predictive factor in a clinical trial involving 58 patients with advanced NSCLC and that patients with low COL4A3-expressing tumors had considerably longer median OS than those with high COL4A3-expressing tumors (44). According to Liu et al., COL5A1 was discovered to be substantially expressed in patients with lung adenocarcinoma who had short survival times and recurrences. COL5A1 knockdown in metastatic human adenocarcinoma cells also hindered cell growth and invasion and brought on apoptosis (45).

Collagen, a significant part of the extracellular matrix, was traditionally believed to play a passive role in the development of tumors. In this study, collagen signature was correlated with TAM infiltration and cell type. However, it is not clear how ECM regulates TAM infiltration (46). Recent studies have shown that collagen degradation products have chemotactic effects on immune cell infiltration (47–49). The COL6 ETP peptide was found to promote tumor inflammation by increasing macrophage recruitment and upregulating inflammatory factors such as IL-6 and TNF-α (47). Madeleine et al. found that collagen entered TAMs *via* mannose receptor endocytosis and was subsequently degraded by lysosomes, resulting in macrophage phenotype reprogramming and intratumoral fibrosis in pancreatic cancer (48). In a study that mimicked TAM differentiation in tumors *in vitro*, high collagen density in tumors was found to directly affect the transcriptional profile of macrophages, leading to the acquisition of an immunosuppressive phenotype by macrophages. This phenotype suppressed tumor-infiltrating T cell activity and thus reduced the efficacy of immunotherapy (49). Therefore, collagen metabolism and remodeling may affect tumor immune cell infiltration.

Many researchers recognize that the interaction between TMB and ECM is important. CAF are host cells that secrete collagen (50). Mutations in CAF or tumor cells directly or indirectly alter the collagen secretion profile. GSEA analysis showed that the P53 signaling pathway was activated in the high-risk group, which suggested that mutations in the P53 might play a role in the development of LUAD with a high-risk collagen profile. Mutation of the P53 gene in cancer cells can cause changes in the composition and structure of extracellular collagen. P53 inactivation increases collagen deposition, structural remodeling and local tumor invasion (51). P53 deletion or mutation in cancer cells, or inhibition of JAK2 or STAT3 activation, reduced fibrotic reaction and the number of pancreatic stellate cells in the pancreatic cancer stroma (52). The secretomes of lung cancer CAFs are altered by epigenetic silencing of the p53 gene, specifically the matrix components secreted, which modifies the behavior of adjacent cancer cells to facilitate invasion (53). In addition, the specific mutation of P53 gene in pancreatic cancer cells can guide local CAF reprogramming and tumor matrix remodeling, and establish of an environment permissive to invasion and metastasis (54).

In addition, we found that the higher risk score was positively correlated with the expression of almost all CAFs markers (**Figure S4**), implying that both collagen-risk signature and CAFs markers may be involved in the formation of the ECM and development. The collagen-risk signature is an assessment indicator based on the expression pattern of 5 collagen in LUAD. These results therefore suggest that there may be a close link between the ECM and the characteristics of the cancers themselves, with important implications for a deeper understanding of LUAD development and for providing new targets for tumor therapy.

The study still has some limitations. First, our study confirms that Collagen-Risk score can be a valid independent prognostic factor. However, this conclusion is based on 6 retrospective data sets, and future prospective studies are needed to confirm the validity of this conclusion. As this study analyses transcriptomic data from different populations, different testing platforms, there are inevitably some biases in this study. Second, the clinical data we extracted from the TCGA and GEO databases are incomplete. For example, we were unable to successfully extract smoking status and analyze the correlation between smoking status and collagen signature. Finally, we performed laboratory validation using recently collected tumor tissue and normal tissue from our institution, however, we were unable to collect survival information and clinical data on these samples for validation of our prognostic model.

In summary, we identified a reliable prognostic collagen signature based on TCGA database. We present for the first time the prognostic model based on collagen family members and describe in detail the relationship between the collagen signature and the tumor microenvironment, which may provide prognostic information for immunotherapy in patients with LUAD. These significant new discoveries will enable clinicians to treat LUAD patients more individually.

## Data availability statement

The datasets presented in this study can be found in online repositories. The names of the repository/repositories and accession number(s) can be found in the article/**Supplementary Material**.

## Ethics statement

All study protocols were approved by the Ethics Committee of Shaoxing People's Hospital (in accordance with the Declaration of Helsinki). All patients signed a written informed consent for the study and publication.

## Author contributions

GY, LD, and LF conceived and designed the study. TZ and ZL contributed to the acquisition of data. JD, JJZ and JDZ analyzed and interpreted the data. LD wrote the manuscript. XW and YW reviewed and edited the manuscript. All authors reviewed and gave final approval of the manuscript.

## References

[1] Sung H Ferlay J Siegel RL Laversanne M Seorjomataram I Jemal F. Global cancer statistics 2020: GLOBOCAN estimates of incidence and mortality worldwide for 36 cancers in 185 countries. CA Cancer J Clin (2021) 71(3):209–49. doi: 10.3322/caac.21660 33538338

[2] Siegel RL Miller KD Fuchs HE Jemal A. Cancer statistics, 2022. CA Cancer J Clin (2022) 72(1):7–33. doi: 10.3322/caac.21708 35020204

[3] Abe Y Tanaka N. The hedgehog signaling networks in lung cancer: the mechanisms and roles in tumor progression and implications for cancer therapy. BioMed Res Int (2016) 2016:7969286. doi: 10.1155/2016/7969286 28105432 PMC5220431

[4] Hirsch FR Scagliotti GV Mulshine JL Kwon R Jr W JC Wu YL. Lung cancer: current therapies and new targeted treatments. Lancet (2017) 389(10066):299–311. doi: 10.1016/S0140-6736(16)30958-8 27574741

[5] Daigo Y Nakamura Y. From cancer genomics to thoracic oncology: discovery of new biomarkers and therapeutic targets for lung and esophageal carcinoma. Gen Thorac Cardiovasc Surg (2008) 56(2):43–53. doi: 10.1007/s11748-007-0211-x 18297458

[6] Chen P Liu Y Wen Y Zhou C. Non-small cell lung cancer in China [J]. Cancer Commun (Lond) (2022) 42(10):937–70. doi: 10.1002/cac2.12359 PMC9558689 36075878

[7] Valdoz JC Johnson BC Jacobs DJ Franks NA Dodson EL Sanders C. The ECM: to scaffold, or not to scaffold, that is the question. Int J Mol Sci (2021) 22(23):12690. doi: 10.3390/ijms222312690 34884495 PMC8657545

[8] Chaffer CL Weinberg RA. A perspective on cancer cell metastasis. Science (2011) 331(6024):1559–64. doi: 10.1126/science.1203543 21436443

[9] Venning FA Wullkopf L Erler JT. Targeting ECM disrupts cancer progression. Front Oncol (2015) 5. doi: 10.3389/fonc.2015.00224 PMC4611145 26539408

[10] Mohan V Das A Sagi I. Emerging roles of ECM remodeling processes in cancer. Semin Cancer Biol (2020) 62:192–200. doi: 10.1016/j.semcancer.2019.09.004 31518697

[11] Ricard-Blum S. The collagen family. Cold Spring Harb Perspect Biol (2011) 3(1):a004978. doi: 10.1101/cshperspect.a004978 21421911 PMC3003457

[12] Xu S Xu H Wang W Li S Hao L Li T. The role of collagen in cancer: from bench to bedside. J Transl Med (2019) 17(1):309. doi: 10.1186/s12967-019-2058-1 31521169 PMC6744664

[13] Gu L Shan T Ma YX Tay FR Niu L. Novel biomedical applications of crosslinked collagen. Trends Biotechnol (2019) 37(5):464–91. doi: 10.1016/j.tibtech.2018.10.007 30447877

[14] Fang M Yuan J Peng C Tay FR Niu L. Collagen as a double-edged sword in tumor progression. Tumour Biol (2014) 35(4):2871–82. doi: 10.1007/s13277-013-1511-7 PMC3980040 24338768

[15] Amenta PS Briggs K Xu K Gamboa E Jukkola AF Li D. Type XV collagen in human colonic adenocarcinomas has a different distribution than other basement membrane zone proteins. Hum Pathol (2000) 31(3):359–66. doi: 10.1016/S0046-8177(00)80251-8 10746680

[16] Amenta PS Hadad S Lee MT Barnard N Li D Myers JC. Loss of types XV and XIX collagen precedes basement membrane invasion in ductal carcinoma of the female breast. J Pathol (2003) 199(3):298–308. doi: 10.1002/path.1303 12579531

[17] Newman AM Liu CL Green MR Gentles AJ Feng W Xu Y. Robust enumeration of cell subsets from tissue expression profiles. Nat Methods (2015) 12(5):453–7. doi: 10.1038/nmeth.3337 PMC4739640 25822800

[18] Yoshihara K Shahmoradgoli M Martinez E Vegesna R Kim H Garcia WT. Inferring tumour purity and stromal and immune cell admixture from expression data. Nat Commun (2013) 4:2612. doi: 10.1038/ncomms3612 24113773 PMC3826632

[19] Liu LP Lu L Zhao QQ Kou QJ Jiamg ZZ Gui R. Identification and validation of the pyroptosis-related molecular subtypes of lung adenocarcinoma by bioinformatics and machine learning. Front Cell Dev Biol (2021) 9. doi: 10.3389/fcell.2021.756340 PMC8599430 34805165

[20] Wood SL Pernemalm M Crosbie PA Whetton AD. The role of the tumor-microenvironment in lung cancer-metastasis and its relationship to potential therapeutic targets. Cancer Treat Rev (2014) 40(4):558–66. doi: 10.1016/j.ctrv.2013.10.001 24176790

[21] Pan Y Yu Y Wang X Zhang T. Tumor-associated macrophages in tumor immunity. Front Immunol (2020) 11. doi: 10.3389/fimmu.2020.583084 PMC7751482 33365025

[22] Cassetta L Pollard JW. Targeting macrophages: therapeutic approaches in cancer. Nat Rev Drug Discovery (2018) 17(12):887–904. doi: 10.1038/nrd.2018.169 30361552

[23] Najafi M Hashemi Goradel N Farhood B Farhood B Salehi E Nashtaei MS. Macrophage polarity in cancer: a review. J Cell Biochem (2019) 120(3):2756–65. doi: 10.1002/jcb.27646 30270458

[24] Li X Liu R Su X Pan Y Han X Shao C. Harnessing tumor-associated macrophages as aids for cancer immunotherapy. Mol Cancer (2019) 18(1):177. doi: 10.1186/s12943-019-1102-3 31805946 PMC6894344

[25] Kalluri R Zeisberg M. Fibroblasts in cancer. Nat Rev Cancer (2006) 6(5):392–401. doi: 10.1038/nrc1877 16572188

[26] Zhao Z Li T Yuan Y Zhu Y. What is new in cancer-associated fibroblast biomarkers? Cell Commun Signal (2023) 21(1):96. doi: 10.1186/s12964-023-01125-0 37143134 PMC10158035

[27] Havel JJ Chowell D Chan TA. The evolving landscape of biomarkers for checkpoint inhibitor immunotherapy. Nat Rev Cancer (2019) 19(3):133–50. doi: 10.1038/s41568-019-0116-x PMC6705396 30755690

[28] Reck M Remon J Hellmann MD. First-line immunotherapy for non-Small-Cell lung cancer. J Clin Oncol (2022) 40(6):586–97. doi: 10.1200/JCO.21.01497 34985920

[29] Duan H Wang T Luo Z Tong L Dong X Zhang Y. Neoadjuvant programmed cell death protein 1 inhibitors combined with chemotherapy in resectable non-small cell lung cancer: an open-label, multicenter, single-arm study. Transl Lung Cancer Res (2021) 10(2):1020–8. doi: 10.21037/tlcr-21-130 PMC7947385 33718040

[30] Provencio M Nadal E Insa A Campelo M RG Rubio JC Domine M. Neoadjuvant chemotherapy and nivolumab in resectable non-small-cell lung cancer (NADIM): an open-label, multicentre, single-arm, phase 2 trial. Lancet Oncol (2020) 21(11):1413–22. doi: 10.1016/S1470-2045(20)30453-8 32979984

[31] Provencio M Serna-Blasco R Nadal E Insa A Campelo MRG Rubio JC. Overall survival and biomarker analysis of neoadjuvant nivolumab plus chemotherapy in operable stage IIIA non-Small-Cell lung cancer (NADIM phase II trial). J Clin Oncol (2022) 40(25):2924–33. doi: 10.1200/JCO.21.02660 PMC9426809 35576508

[32] Tian S Fu L Zhang J Xu J Qin J Zhang W. Identification of a DNA methylation-driven genes-based prognostic model and drug targets in breast cancer: in silico screening of therapeutic compounds and *in vitro* characterization. Front Immunol (2021) 12. doi: 10.3389/fimmu.2021.761326 PMC8567755 34745136

[33] Spella M Stathopoulos GT. Immune resistance in lung adenocarcinoma. Cancers (Basel) (2021) 13(3):384. doi: 10.3390/cancers13030384 33494181 PMC7864325

[34] Liu XS Zhou LM Yuan LL Gao Y Kui XY Liu XY. NPM1 is a prognostic biomarker involved in immune infiltration of lung adenocarcinoma and associated with m6A modification and glycolysis. Front Immunol (2021) 12. doi: 10.3389/fimmu.2021.724741 PMC8324208 34335635

[35] Tian Q Zhou Y Zhu L Gao H Yang J. Development and validation of a ferroptosis-related gene signature for overall survival prediction in lung adenocarcinoma. Front Cell Dev Biol (2021) 9. doi: 10.3389/fcell.2021.684259 PMC8294813 34307361

[36] Thai AA Solomon BJ Sequist LV Gainor JF Heist RS. Lung cancer. Lancet (2021) 398(10299):535–54. doi: 10.1016/S0140-6736(21)00312-3 34273294

[37] Cox TR. The matrix in cancer. Nat Rev Cancer (2021) 21(4):217–38. doi: 10.1038/s41568-020-00329-7 33589810

[38] Hou L Lin T Wang Y Liu B Wang M. Collagen type 1 alpha 1 chain is a novel predictive biomarker of poor progression-free survival and chemoresistance in metastatic lung cancer. J Cancer (2021) 12(19):5723–31. doi: 10.7150/jca.59723 PMC8408119 34475986

[39] Dong S Zhu P Zhang S. Expression of collagen type 1 alpha 1 indicates lymph node metastasis and poor outcomes in squamous cell carcinomas of the lung. PeerJ (2020) 8:e10089. doi: 10.7717/peerj.10089 33062455 PMC7531356

[40] Fang S Dai Y Mei Y Yang M Hu L Yang H. Clinical significance and biological role of cancer-derived type I collagen in lung and esophageal cancers. Thorac Cancer (2019) 10(2):277–88. doi: 10.1111/1759-7714.12947 PMC6360244 30604926

[41] Chong IW Chang MY Chang HC Yu YP Sheu CC Tsai JR. Great potential of a panel of multiple hMTH1, SPD, ITGA11 and COL11A1 markers for diagnosis of patients with non-small cell lung cancer. Oncol Rep (2006) 16(5):981–8. doi: 10.3892/or.16.5.981 17016581

[42] Shen L Yang M Lin Q Zhang Z Zhu B Miao C. COL11A1 is overexpressed in recurrent non-small cell lung cancer and promotes cell proliferation, migration, invasion and drug resistance. Oncol Rep (2016) 36(2):877–85. doi: 10.3892/or.2016.4869 27373316

[43] Weng TY Wang CY Hung YH Chen WC Chen YL Lai MD. Differential expression pattern of THBS1 and THBS2 in lung cancer: clinical outcome and a systematic-analysis of microarray databases. PloS One (2016) 11(8):e0161007. doi: 10.1371/journal.pone.0161007 27513329 PMC4981437

[44] Jiang CP Wu BH Chen SP Fu MY Yang M Liu F. High COL4A3 expression correlates with poor prognosis after cisplatin plus gemcitabine chemotherapy in non-small cell lung cancer. Tumour Biol (2013) 34(1):415–20. doi: 10.1007/s13277-012-0565-2 23108892

[45] Liu W Wei H Gao Z Chen G Liu Y Gao X. COL5A1 may contribute the metastasis of lung adenocarcinoma. Gene (2018) 665:57–66. doi: 10.1016/j.gene.2018.04.066 29702185

[46] Khalaf K Hana D Chou JT Singh C Mackiewicz A Kaczmarek M. Aspects of the tumor microenvironment involved in immune resistance and drug resistance. Front Immunol (2021) 12:656364. doi: 10.3389/fimmu.2021.656364 34122412 PMC8190405

[47] Wang J Pan W. The biological role of the collagen alpha-3 (VI) chain and its cleaved C5 domain fragment endotrophin in cancer. Onco Targets Ther (2020) 13:5779–93. doi: 10.2147/OTT.S256654 PMC7319802 32606789

[48] Larue MM Parker S Puccini J Cammer M Kimmelman AC Sagi DB. Metabolic reprogramming of tumor-associated macrophages by collagen turnover promotes fibrosis in pancreatic cancer. Proc Natl Acad Sci USA (2022) 119(16):e2119168119. doi: 10.1073/pnas.2119168119 35412885 PMC9169723

[49] Larsen AMH Kuczek DE Kalvisa A Kalvisa A Siersbækm S Thorseth M L. Collagen density modulates the immunosuppressive functions of macrophages. J Immunol (2020) 205(5):1461–72. doi: 10.4049/jimmunol.1900789 32839214

[50] Rasanen K Vaheri A. Activation of fibroblasts in cancer stroma. Exp Cell Res (2010) 316(17):2713–22. doi: 10.1016/j.yexcr.2010.04.032 20451516

[51] Kenny TC Schmidt H Adelson K Hoshida Y Koh A P Shah N. Patient-derived interstitial fluids and predisposition to aggressive sporadic breast cancer through collagen remodeling and inactivation of p53. Clin Cancer Res (2017) 23(18):5446–59. doi: 10.1158/1078-0432.CCR-17-0342 PMC5600839 28630214

[52] Wormann SM Song L Ai J Diakopoulos K N Kurkowski M U Gorgulu K. Loss of P53 function activates JAK2-STAT3 signaling to promote pancreatic tumor growth, stroma modification, and gemcitabine resistance in mice and is associated with patient survival. Gastroenterology (2016) 151(1):180–93.e12. doi: 10.1053/j.gastro.2016.03.010 27003603

[53] Arandkar S Furth N Elisha Y Nataraj NB Kuip HVD Yarden Y. Altered p53 functionality in cancer-associated fibroblasts contributes to their cancer-supporting features. Proc Natl Acad Sci USA (2018) 115(25):6410–5. doi: 10.1073/pnas.1719076115 PMC6016816 29866855

[54] Vennin C Melenec P Rouet R Nobis M Cazet AS Murphy KJ. CAF hierarchy driven by pancreatic cancer cell p53-status creates a pro-metastatic and chemoresistant environment *via* perlecan. Nat Commun (2019) 10(1):3637. doi: 10.1038/s41467-019-10968-6 31406163 PMC6691013

