# Supplementary material for: A five-collagen-based risk model in lung adenocarcinoma: prognostic significance and immune landscape
Source: Front Oncol. 2023 Jul 5;13:1180723. doi: 10.3389/fonc.2023.1180723 (PMC10354438; doi:10.3389/fonc.2023.1180723)
Supplement: Supplementary file 1 [file DataSheet_1.pdf]

Table S1: Clinical characteristics of LUAD from 6 cohorts.

Characteristic	TCGA(N=522)	GSE11969(N=149)	GSE13213(N=117)	GSE30219(N=85)	GSE31210(N=226)	GSE72094(N=420)
**Age**						
≤65	214(46.17%)	62(41.61%)	78(66.67%)	60(70.59%)	176(77.88%)	127(30.24%)
>65	262(50.19%)	87(58.39%)	39(33.33%)	25(29.41%)	50(22.12%)	293(69.76%)
unknown	19(3.64%)	0	0	0	0	0
**Gender**						
Female	280(53.64%)	48(32.43%)	57 (48.7%)	19 (22.4%)	121 (53.54%)	232 (55.24%)
Male	242(46.36%)	101(67.57%)	60 (51.3%)	66 (77.6%)	105 (46.46%)	188 (44.76%)
unknown	-	0	0	0	0	0
**Stage**						
I/ II	403(77.20%)	104(69.80%)	92 (78.6%)	84(98.82%)	226 (100.00%)	334 (79.52%)
III/ IV	111(21.26%)	45(30.20%)	25 (21.4%)	1(1.18%)	0	80 (19.05%)
unknown	8(1.53%)	0	0	0	0	6 (1.43%)
**T classification**						
T1	172(32.95%)	50(33.55%)	54(46.15%)	71(83.53%)	-	-
T2	281(53.83%)	73(49.00%)	50(42.74%)	12(14.12%)	-	-
T3	47(9.00%)	18(12.08%)	8(6.84%)	2(2.35%)	-	-
T4	19(3.64%)	8(5.37%)	5(4.27%)	0	-	-
unknown	3(0.57%)	0	0	0	-	-
**N classification**						
N0	335(64.18%)	93(62.42%)	87(74.36%)	82(96.47%)	-	-
N1	98(18.77%)	21(14.09%)	8(6.84%)	3(3.53%)	-	-
N2	75(14.37%)	33(22.15%)	22(18.80%)	0	-	-
N3	2(0.38%)	2(1.34%)	0	0	-	-

**Characteristic**	**TCGA(N=522)**	**GSE11969(N=149)**	**GSE13213(N=117)**	**GSE30219(N=85)**	**GSE31210(N=226)**	**GSE72094(N=420)**
unknown	12(2.30%)	0	0	0	-	-
**M classification**						
M0	353(67.62%)	149(100.00%)	117(100.00%)	85(100.00%)	-	-
M1	25(4.79%)	0	0	0	-	-
unknown	144(27.59%)	0	0	0	-	-
**Status**						
Alive	355(68.01%)	75(50.34%)	68 (58.1%)	40 (47.05%)	191 (84.51%)	298 (70.95%)
Death	167(37.99%)	74(49.66%)	49 (41.9%)	45 (52.95%)	35 (15.49%)	122 (29.05%)

Table S2: Primer sequences for quantitative real-time polymerase chain reaction.

Primer name	Sequence (5'-3')
COL1A1	F: GAGGGCCAAGACGAAGACATC
	R: CAGATCACGTCATCGCACAAC
COL4A3	F: AGCAAGGGTTGTGTCTGTAAAG
	R: CAGAAAATCCTGGCAATCCACT
COL5A1	F: GCCCGGATGTCGCTTACAG
	R: AAATGCAGACGCAGGGTACAG
COL11A1	F: ACCCTCGCATTGACCTTCC
	R: TTTGTGCAAAATCCCGTTGTTT
COL22A1	F: CCTAGCGTTCGTGTAGAAGGA
	R: CCCATCCGTACATAGGAACTCT

Table S3: GO functional enrichment of correlated genes.

Description	GeneRatio	BgRatio	P value	P adjust	q value	Count	Gene ID	Group
extracellular matrix organization	13/52	334/17910	8.63E-12	1.27E-08	1.03E-08	13	COL4A3/COL4A4/COL5A1/SULF2/SULF1/COL1A2/COL12A1/LOXL2/COL5A2/COL11A1/MMP14/ITGA5/ADAM12	BP
extracellular structure organization	13/52	387/17910	5.43E-11	3.99E-08	3.24E-08	13	COL4A3/COL4A4/COL5A1/SULF2/SULF1/COL1A2/COL12A1/LOXL2/COL5A2/COL11A1/MMP14/ITGA5/ADAM12	BP
collagen fibril organization	6/52	45/17910	3.32E-09	1.22E-06	9.91E-07	6	COL5A1/COL1A2/COL12A1/LOXL2/COL5A2/COL11A1	BP
endodermal cell differentiation	6/52	45/17910	3.32E-09	1.22E-06	9.91E-07	6	COL5A1/COL12A1/COL5A2/COL11A1/MMP14/ITGA5	BP
endoderm formation	6/52	50/17910	6.41E-09	1.89E-06	1.53E-06	6	COL5A1/COL12A1/COL5A2/COL11A1/MMP14/ITGA5	BP
glomerular basement membrane development	4/52	10/17910	1.31E-08	3.21E-06	2.60E-06	4	COL4A3/COL4A4/SULF2/SULF1	BP
endoderm development	6/52	73/17910	6.53E-08	1.37E-05	1.11E-05	6	COL5A1/COL12A1/COL5A2/COL11A1/MMP14/ITGA5	BP
formation of primary germ layer	6/52	117/17910	1.09E-06	0.000178	0.000144	6	COL5A1/COL12A1/COL5A2/COL11A1/MMP14/ITGA5	BP
embryonic skeletal system development	6/52	117/17910	1.09E-06	0.000178	0.000144	6	TWIST1/PRRX1/SULF2/SULF1/COL11A1/MMP14	BP
anatomical structure morphogenesis	21/52	2535/17910	3.10E-06	0.000456	0.00037	21	COL4A3/SFTPB/THBS2/COL5A1/TWIST1/PRRX1/SULF2/SULF1/COL1A2/COL12A1/GPC6/FRMD6/LOXL2/COL5A2/COL11A1/POSTN/ADAMTS12/MMP14/ACTN1/ITGA5/ADAM12	BP
connective tissue development	7/52	243/17910	6.17E-06	0.000826	0.000669	7	COL5A1/PRRX1/SULF2/SULF1/LOXL2/COL11A1/ADAMTS12	BP
movement of cell or subcellular component	18/52	2012/17910	7.44E-06	0.000875	0.000709	18	ATP8A1/GPD1L/COL5A1/TWIST1/MAP4K4/SLC16A1/SULF1/COL1A2/TNFAIP6/GPC6/FRMD6/LOXL2/POSTN/ADAMTS12/MMP14/ACTN1/ITGA5/SPOCK1	BP

blood vessel development	11/52	751/17910	8.20E-06	0.000875	0.000709	11	COL4A3/THBS2/COL5A1/TWIST1/PRRX1/SULF1/COL1A2/LOXL2/MMP14/ITGA5/ADAM12	BP
skeletal system development	9/52	479/17910	8.60E-06	0.000875	0.000709	9	TWIST1/PRRX1/SULF2/SULF1/COL1A2/LOXL2/COL11A1/ADAMTS12/MMP14	BP
chondrocyte differentiation	5/52	97/17910	8.92E-06	0.000875	0.000709	5	SULF2/SULF1/LOXL2/COL11A1/ADAMTS12	BP
cell migration	15/52	1474/17910	1.21E-05	0.000981	0.000795	15	ATP8A1/COL5A1/TWIST1/MAP4K4/SLC16A1/SULF1/COL1A2/TNFAIP6/GPC6/LOXL2/POSTN/ADAMTS12/MMP14/ITGA5/SPOCK1	BP
vasculature development	11/52	784/17910	1.23E-05	0.000981	0.000795	11	COL4A3/THBS2/COL5A1/TWIST1/PRRX1/SULF1/COL1A2/LOXL2/MMP14/ITGA5/ADAM12	BP
gastrulation	6/52	178/17910	1.23E-05	0.000981	0.000795	6	COL5A1/COL12A1/COL5A2/COL11A1/MMP14/ITGA5	BP
cardiovascular system development	11/52	792/17910	1.35E-05	0.000981	0.000795	11	COL4A3/THBS2/COL5A1/TWIST1/PRRX1/SULF1/COL1A2/LOXL2/MMP14/ITGA5/ADAM12	BP
anatomical structure formation involved in morphogenesis	13/52	1124/17910	1.39E-05	0.000981	0.000795	13	COL4A3/THBS2/COL5A1/TWIST1/SULF1/COL12A1/LOXL2/COL5A2/COL11A1/MMP14/ACTN1/ITGA5/ADAM12	BP
cartilage development	6/52	182/17910	1.40E-05	0.000981	0.000795	6	PRRX1/SULF2/SULF1/LOXL2/COL11A1/ADAMTS12	BP
cell adhesion	14/52	1338/17910	1.88E-05	0.001256	0.001018	14	COL4A3/THBS2/COL5A1/MAP4K4/COL12A1/TNFAIP6/LOXL2/POSTN/ADAMTS12/MMP14/ACTN1/ITGA5/ADAM12/SPOCK1	BP
biological adhesion	14/52	1345/17910	1.99E-05	0.001274	0.001033	14	COL4A3/THBS2/COL5A1/MAP4K4/COL12A1/TNFAIP6/LOXL2/POSTN/ADAMTS12/MMP14/ACTN1/ITGA5/ADAM12/SPOCK1	BP
negative regulation of endodermal cell	2/52	3/17910	2.48E-05	0.001517	0.00123	2	COL5A1/COL5A2	BP

differentiation								
glomerulus development	4/52	61/17910	2.92E-05	0.001717	0.001392	4	COL4A3/COL4A4/SULF2/SULF1	BP
embryonic morphogenesis	9/52	565/17910	3.18E-05	0.001801	0.00146	9	COL5A1/TWIST1/PRRX1/SULF1/COL12A1/COL5A2/COL11A1/MMP14/ITGA5	BP
cell motility	15/52	1611/17910	3.48E-05	0.001829	0.001483	15	ATP8A1/COL5A1/TWIST1/MAP4K4/SLC16A1/SULF1/COL1A2/TNFAIP6/GPC6/LOXL2/POSTN/ADAMTS12/MMP14/ITGA5/SPOCK1	BP
localization of cell	15/52	1611/17910	3.48E-05	0.001829	0.001483	15	ATP8A1/COL5A1/TWIST1/MAP4K4/SLC16A1/SULF1/COL1A2/TNFAIP6/GPC6/LOXL2/POSTN/ADAMTS12/MMP14/ITGA5/SPOCK1	BP
cell-substrate adhesion	7/52	322/17910	3.80E-05	0.001928	0.001563	7	MAP4K4/POSTN/ADAMTS12/MMP14/ACTN1/ITGA5/SPOCK1	BP
negative regulation of cell-substrate adhesion	4/52	67/17910	4.23E-05	0.002074	0.001682	4	MAP4K4/POSTN/MMP14/SPOCK1	BP
cellular water homeostasis	2/52	4/17910	4.94E-05	0.002345	0.001901	2	AQP4/SCTR	BP
negative regulation of embryonic development	3/52	26/17910	5.73E-05	0.002571	0.002084	3	COL5A1/SULF1/COL5A2	BP
regulation of anatomical structure morphogenesis	12/52	1102/17910	5.77E-05	0.002571	0.002084	12	COL4A3/THBS2/COL5A1/TWIST1/SULF2/SULF1/GPC6/COL5A2/POSTN/ADAMTS12/ITGA5/ADAM12	BP
chondrocyte development	3/52	27/17910	6.43E-05	0.002781	0.002255	3	SULF2/SULF1/COL11A1	BP
circulatory system development	12/52	1128/17910	7.22E-05	0.003036	0.002462	12	COL4A3/THBS2/COL5A1/TWIST1/PRRX1/SULF1/COL1A2/LOXL2/COL11A1/MMP14/ITGA5/ADAM12	BP

proteoglycan metabolic process	4/52	79/17910	8.08E-05	0.003269	0.00265	4	SULF2/SULF1/COL11A1/ADAMTS12	BP
regulation of endodermal cell differentiation	2/52	5/17910	8.22E-05	0.003269	0.00265	2	COL5A1/COL5A2	BP
tissue development	16/52	1964/17910	9.02E-05	0.00349	0.00283	16	COL5A1/TWIST1/PRRX1/SULF2/SULF1/COL1A2/COL12A1/GPC6/FRMD6/LOXL2/COL5A2/COL11A1/POSTN/ADAMTS12/MMP14/ITGA5	BP
carbohydrate derivative metabolic process	12/52	1169/17910	0.000102	0.003789	0.003072	12	ESYT3/GPD1L/CHIA/SULT1A2/ST3GAL5/HS3ST3A1/SULF2/SULF1/POGLUT2/GPC6/COL11A1/ADAMTS12	BP
animal organ morphogenesis	11/52	989/17910	0.000103	0.003789	0.003072	11	SFTPB/COL5A1/TWIST1/PRRX1/SULF2/SULF1/COL1A2/GPC6/COL5A2/COL11A1/MMP14	BP
embryonic skeletal system morphogenesis	4/52	85/17910	0.000108	0.003858	0.003128	4	TWIST1/PRRX1/COL11A1/MMP14	BP
blood vessel morphogenesis	9/52	670/17910	0.000118	0.004145	0.003361	9	COL4A3/THBS2/TWIST1/PRRX1/SULF1/LOXL2/MMP14/ITGA5/ADAM12	BP
negative regulation of developmental process	11/52	1017/17910	0.000132	0.004516	0.003661	11	COL4A3/THBS2/COL5A1/TWIST1/MAP4K4/SULF1/LOXL2/COL5A2/POSTN/ADAMTS12/SPOCK1	BP
complex of collagen trimers	6/58	18/18675	1.24E-11	2.82E-09	2.26E-09	6	COL4A3/COL4A4/COL5A1/COL1A2/COL5A2/COL11A1	CC
extracellular matrix component	7/58	47/18675	1.09E-10	8.90E-09	7.14E-09	7	COL4A3/COL4A4/COL5A1/COL1A2/COL12A1/COL5A2/COL11A1	CC
extracellular matrix	14/58	468/18675	1.18E-10	8.90E-09	7.14E-09	14	COL4A3/COL4A4/THBS2/COL5A1/SULF1/COL1A2/COL12A1/MXRA5/GPC6/LOXL2/COL5A2/COL11A1/POSTN/MMP14	CC
collagen-containing extracellular matrix	13/58	399/18675	2.10E-10	1.05E-08	8.39E-09	13	COL4A3/COL4A4/THBS2/COL5A1/SULF1/COL1A2/COL12A1/MXRA5/GPC6/LOXL2/COL5A2/COL11A1/POSTN	CC

collagen trimer	8/58	86/18675	2.30E-10	1.05E-08	8.39E-09	8	COL4A3/COL4A4/COL5A1/COL1A2/COL12A1/COL5A2/COL11A1/COL22A1	CC
extracellular region	33/58	4295/18675	2.64E-08	7.72E-07	6.19E-07	33	COL4A3/COL4A4/C16orf89/SELENBP1/NAPSA/ATP8A1/GPD1L/SFTPB/SCGB3A2/CHIA/SFTA2/OLFML2B/THBS2/COL5A1/SLC16A1/SULF2/ENOX1/SULF1/COL1A2/COL12A1/MXRA5/TNFAIP6/GPC6/LOXL2/COL5A2/COL11A1/POSTN/ADAMTS12/MMP14/ACTN1/ADAM12/SPOCK1/COL22A1	CC
fibrillar collagen trimer	4/58	11/18675	2.72E-08	7.72E-07	6.19E-07	4	COL5A1/COL1A2/COL5A2/COL11A1	CC
banded collagen fibril	4/58	11/18675	2.72E-08	7.72E-07	6.19E-07	4	COL5A1/COL1A2/COL5A2/COL11A1	CC
endoplasmic reticulum lumen	9/58	306/18675	3.99E-07	1.01E-05	8.07E-06	9	COL4A3/COL4A4/COL5A1/COL1A2/COL12A1/POGLUT2/COL5A2/COL11A1/COL22A1	CC
endoplasmic reticulum	19/58	1785/18675	9.49E-07	2.15E-05	1.73E-05	19	COL4A3/COL4A4/ESYT3/ABCC6/ATP8A1/SFTPB/CYB5A/CYP4B1/COL5A1/SULF2/SULF1/COL1A2/COL12A1/POGLUT2/LOXL2/COL5A2/COL11A1/ITGA5/COL22A1	CC
endomembrane system	29/58	4213/18675	4.25E-06	8.76E-05	7.03E-05	29	COL4A3/COL4A4/ESYT3/ABCC6/NAPSA/ATP8A1/SFTPB/CYB5A/CYP4B1/SFTA2/ST3GAL5/THBS2/COL5A1/HS3ST3A1/SULF2/SULF1/COL1A2/COL12A1/POGLUT2/TNFAIP6/GPC6/LOXL2/COL5A2/COL11A1/POSTN/MMP14/ACTN1/ITGA5/COL22A1	CC
extracellular region part	25/58	3312/18675	6.07E-06	0.000115	9.21E-05	25	COL4A3/COL4A4/C16orf89/SELENBP1/NAPSA/ATP8A1/GPD1L/CHIA/THBS2/COL5A1/SLC16A1/SULF2/ENOX1/SULF1/COL1A2/COL12A1/MXRA5/GPC6/LOXL2/COL5A2/COL11A1/POSTN/MMP14/ACTN1/SPOCK1	CC
basement membrane	5/58	91/18675	9.19E-06	0.00016	0.000129	5	COL4A3/COL4A4/THBS2/COL5A1/LOXL2	CC
collagen type V trimer	2/58	3/18675	2.84E-05	0.000449	0.00036	2	COL5A1/COL5A2	CC
endoplasmic reticulum	14/58	1289/18675	2.97E-05	0.000449	0.00036	14	COL4A3/COL4A4/ESYT3/ABCC6/SFTPB/CYB5A/CYP4B1/C	CC

part							OL5A1/COL1A2/COL12A1/POGLUT2/COL5A2/COL11A1/COL22A1	
collagen type IV trimer	2/58	5/18675	9.42E-05	0.001337	0.001073	2	COL4A3/COL4A4	CC
network-forming collagen trimer	2/58	6/18675	0.000141	0.001779	0.001427	2	COL4A3/COL4A4	CC
collagen network	2/58	6/18675	0.000141	0.001779	0.001427	2	COL4A3/COL4A4	CC
multivesicular body lumen	2/58	7/18675	0.000197	0.002237	0.001795	2	NAPSA/SFTPB	CC
basement membrane collagen trimer	2/58	7/18675	0.000197	0.002237	0.001795	2	COL4A3/COL4A4	CC
late endosome lumen	2/58	9/18675	0.000337	0.003638	0.002918	2	NAPSA/SFTPB	CC
lamellar body	2/58	17/18675	0.001251	0.01291	0.010357	2	NAPSA/SFTPB	CC
extracellular space	18/58	3051/18675	0.004008	0.039555	0.031732	18	C16orf89/SELENBP1/NAPSA/ATP8A1/GPD1L/CHIA/SLC16A1/SULF2/ENOX1/SULF1/COL1A2/COL12A1/MXRA5/GPC6/LOXL2/MMP14/ACTN1/SPOCK1	CC
supramolecular polymer	8/58	851/18675	0.004577	0.041849	0.033573	8	COL4A3/COL4A4/SCTR/COL5A1/COL1A2/COL5A2/COL11A1/ACTN1	CC
supramolecular complex	8/58	852/18675	0.004609	0.041849	0.033573	8	COL4A3/COL4A4/SCTR/COL5A1/COL1A2/COL5A2/COL11A1/ACTN1	CC
endosome lumen	2/58	34/18675	0.004989	0.043561	0.034946	2	NAPSA/SFTPB	CC
multivesicular body	2/58	41/18675	0.007192	0.060467	0.048508	2	NAPSA/SFTPB	CC
glycerol-3-phosphate dehydrogenase complex	1/58	3/18675	0.009289	0.070286	0.056385	1	GPD1L	CC
anchoring collagen complex	1/58	3/18675	0.009289	0.070286	0.056385	1	COL12A1	CC
alphav-beta3	1/58	3/18675	0.009289	0.070286	0.056385	1	ITGA5	CC

integrin-vitronectin complex								
Golgi apparatus	10/58	1441/18675	0.012541	0.091831	0.073669	10	ATP8A1/SFTA2/ST3GAL5/HS3ST3A1/SULF2/SULF1/GPC6/POSTN/MMP14/ITGA5	CC
neuromuscular junction	2/58	60/18675	0.014951	0.103046	0.082667	2	POSTN/SPOCK1	CC
alveolar lamellar body	1/58	5/18675	0.015434	0.103046	0.082667	1	NAPSA	CC
astrocyte end-foot	1/58	5/18675	0.015434	0.103046	0.082667	1	AQP4	CC
FACIT collagen trimer	1/58	6/18675	0.018493	0.116608	0.093546	1	COL12A1	CC
chromaffin granule membrane	1/58	6/18675	0.018493	0.116608	0.093546	1	ATP8A1	CC
Golgi apparatus part	7/58	891/18675	0.019901	0.122095	0.097948	7	ST3GAL5/HS3ST3A1/SULF2/SULF1/GPC6/POSTN/MMP14	CC
organelle subcompartment	10/58	1592/18675	0.023659	0.132861	0.106584	10	ESYT3/ABCC6/SFTPB/CYB5A/CYP4B1/ST3GAL5/HS3ST3A1/SULF2/SULF1/POSTN	CC
external side of plasma membrane	3/58	199/18675	0.023955	0.132861	0.106584	3	AQP4/ENOX1/ITGA5	CC
chromaffin granule	1/58	8/18675	0.024582	0.132861	0.106584	1	ATP8A1	CC
pinosome	1/58	8/18675	0.024582	0.132861	0.106584	1	MMP14	CC
macropinosome	1/58	8/18675	0.024582	0.132861	0.106584	1	MMP14	CC
side of membrane	4/58	359/18675	0.025264	0.133373	0.106995	4	ESYT3/AQP4/ENOX1/ITGA5	CC
fascia adherens	1/58	10/18675	0.030634	0.158045	0.126788	1	ACTN1	CC
platelet alpha granule	2/58	91/18675	0.032541	0.164152	0.131687	2	THBS2/ACTN1	CC
focal adhesion	4/58	397/18675	0.034729	0.166484	0.133558	4	MAP4K4/MMP14/ACTN1/ITGA5	CC
cell-substrate adherens junction	4/58	399/18675	0.035278	0.166484	0.133558	4	MAP4K4/MMP14/ACTN1/ITGA5	CC
intrinsic component of plasma membrane	8/58	1231/18675	0.035551	0.166484	0.133558	8	ESYT3/VIPR1/AQP4/ST3GAL5/SLC16A1/GPC6/MMP14/ITGA5	CC

astrocyte projection	1/58	12/18675	0.03665	0.166484	0.133558	1	AQP4	CC
cell-substrate junction	4/58	404/18675	0.03667	0.166484	0.133558	4	MAP4K4/MMP14/ACTN1/ITGA5	CC
secretory granule	6/58	806/18675	0.038325	0.170582	0.136846	6	NAPSA/ATP8A1/SFTPB/THBS2/TNFAIP6/ACTN1	CC
Golgi lumen	2/58	102/18675	0.04006	0.174875	0.140289	2	GPC6/MMP14	CC
node of Ranvier	1/58	15/18675	0.045604	0.195311	0.156684	1	SPOCK1	CC
supramolecular fiber	6/58	845/18675	0.046462	0.195311	0.156684	6	SCTR/COL5A1/COL1A2/COL5A2/COL11A1/ACTN1	CC
extracellular matrix structural constituent conferring tensile strength	7/51	41/16967	3.00E-11	3.88E-09	2.91E-09	7	COL4A3/COL4A4/COL5A1/COL1A2/COL12A1/COL5A2/COL11A1	MF
extracellular matrix structural constituent	10/51	158/16967	3.39E-11	3.88E-09	2.91E-09	10	COL4A3/COL4A4/THBS2/COL5A1/COL1A2/COL12A1/MXRA5/COL5A2/COL11A1/POSTN	MF
structural molecule activity	11/51	653/16967	2.95E-06	0.000225	0.000169	11	COL4A3/COL4A4/THBS2/COL5A1/COL1A2/COL12A1/MXRA5/COL5A2/COL11A1/POSTN/ACTN1	MF
integrin binding	5/51	113/16967	2.21E-05	0.001215	0.00091	5	COL4A3/COL5A1/MMP14/ACTN1/ITGA5	MF
N-acetylglucosamine-6-sulfatase activity	2/51	3/16967	2.65E-05	0.001215	0.00091	2	SULF2/SULF1	MF
glycosaminoglycan binding	5/51	199/16967	0.00032	0.012212	0.00915	5	THBS2/COL5A1/TNFAIP6/COL11A1/POSTN	MF
platelet-derived growth factor binding	2/51	11/16967	0.000479	0.015665	0.011737	2	COL5A1/COL1A2	MF
arylsulfatase activity	2/51	12/16967	0.000574	0.016417	0.0123	2	SULF2/SULF1	MF
metalloendopeptidase inhibitor activity	2/51	14/16967	0.000788	0.020043	0.015017	2	COL4A3/SPOCK1	MF
sulfuric ester hydrolase activity	2/51	15/16967	0.000907	0.020774	0.015565	2	SULF2/SULF1	MF

heparin binding	4/51	148/16967	0.00101	0.021034	0.01576	4	THBS2/COL5A1/COL11A1/POSTN	MF
metalloendopeptidase activity	3/51	89/16967	0.002422	0.046225	0.034634	3	ADAMTS12/MMP14/ADAM12	MF
sulfur compound binding	4/51	221/16967	0.004337	0.076395	0.057239	4	THBS2/COL5A1/COL11A1/POSTN	MF
peptide hormone binding	2/51	38/16967	0.005811	0.095058	0.071223	2	VIPR1/SCTR	MF
protein-lysine 6-oxidase activity	1/51	3/16967	0.008991	0.110259	0.082612	1	LOXL2	MF
phosphotransferase activity, nitrogenous group as acceptor	1/51	3/16967	0.008991	0.110259	0.082612	1	MAP4K4	MF
flavonol 3-sulfotransferase activity	1/51	3/16967	0.008991	0.110259	0.082612	1	SULT1A2	MF
heparan sulfate binding	1/51	3/16967	0.008991	0.110259	0.082612	1	COL11A1	MF
sulfotransferase activity	2/51	48/16967	0.009148	0.110259	0.082612	2	SULT1A2/HS3ST3A1	MF
cell adhesion molecule binding	5/51	444/16967	0.010491	0.119017	0.089174	5	COL4A3/COL5A1/MMP14/ACTN1/ITGA5	MF
metallopeptidase activity	3/51	153/16967	0.010914	0.119017	0.089174	3	ADAMTS12/MMP14/ADAM12	MF
calcium ion binding	6/51	635/16967	0.011565	0.120382	0.090197	6	THBS2/SULF2/SULF1/LOXL2/ACTN1/SPOCK1	MF
lactate transmembrane transporter activity	1/51	5/16967	0.014941	0.134047	0.100435	1	SLC16A1	MF
oxidoreductase activity, acting on a sulfur group	1/51	5/16967	0.014941	0.134047	0.100435	1	SELENBP1	MF

of donors, oxygen as acceptor								
protein xylosyltransferase activity	1/51	5/16967	0.014941	0.134047	0.100435	1	POGLUT2	MF
transferase activity, transferring sulfur-containing groups	2/51	63/16967	0.015392	0.134047	0.100435	2	SULT1A2/HS3ST3A1	MF
endopeptidase activity	4/51	322/16967	0.015805	0.134047	0.100435	4	NAPSA/ADAMTS12/MMP14/ADAM12	MF
catalytic activity	22/51	4828/16967	0.017454	0.13512	0.101239	22	ATP13A4/SELENBP1/NAPSA/ATP8A1/CYB5A/GGTLC1/CYP4B1/CHIA/SULT1A2/PLA2G4F/CDKL2/ST3GAL5/HS3ST3A1/MAP4K4/SULF2/ENOX1/SULF1/POGLUT2/LOXL2/ADAMTS12/MMP14/ADAM12	MF
glutathione hydrolase activity	1/51	6/16967	0.017903	0.13512	0.101239	1	GGTLC1	MF
coreceptor activity involved in Wnt signaling pathway, planar cell polarity pathway	1/51	6/16967	0.017903	0.13512	0.101239	1	GPC6	MF
peptidase activity, acting on L-amino acid peptides	5/51	511/16967	0.018291	0.13512	0.101239	5	NAPSA/GGTLC1/ADAMTS12/MMP14/ADAM12	MF
SMAD binding	2/51	72/16967	0.019803	0.140469	0.105247	2	COL1A2/COL5A2	MF
aryl sulfotransferase	1/51	7/16967	0.020856	0.140469	0.105247	1	SULT1A2	MF

activity								
selenium binding	1/51	7/16967	0.020856	0.140469	0.105247	1	SELENBP1	MF
peptidase activity	5/51	536/16967	0.021985	0.143847	0.107778	5	NAPSA/GGTLC1/ADAMTS12/MMP14/ADAM12	MF
metal ion binding	19/51	4068/16967	0.023319	0.147303	0.110367	19	ATP13A4/ATP8A1/CYB5A/CYP4B1/THBS2/COL5A1/SULF2/SULF1/COL1A2/LOXL2/COL5A2/COL11A1/POSTN/ADAMTS12/MMP14/ACTN1/ITGA5/ADAM12/SPOCK1	MF
vascular endothelial growth factor receptor 2 binding	1/51	8/16967	0.0238	0.147303	0.110367	1	ITGA5	MF
hormone binding	2/51	81/16967	0.024677	0.148711	0.111422	2	VIPR1/SCTR	MF
water channel activity	1/51	9/16967	0.026736	0.14882	0.111504	1	AQP4	MF
heparan sulfate sulfotransferase activity	1/51	9/16967	0.026736	0.14882	0.111504	1	HS3ST3A1	MF
coreceptor activity involved in Wnt signaling pathway	1/51	9/16967	0.026736	0.14882	0.111504	1	GPC6	MF
cation binding	19/51	4133/16967	0.027295	0.14882	0.111504	19	ATP13A4/ATP8A1/CYB5A/CYP4B1/THBS2/COL5A1/SULF2/SULF1/COL1A2/LOXL2/COL5A2/COL11A1/POSTN/ADAMTS12/MMP14/ACTN1/ITGA5/ADAM12/SPOCK1	MF
water transmembrane transporter activity	1/51	10/16967	0.029663	0.149222	0.111805	1	AQP4	MF
structural constituent of postsynapse	1/51	10/16967	0.029663	0.149222	0.111805	1	ACTN1	MF
vascular endothelial growth factor receptor binding	1/51	11/16967	0.032581	0.149222	0.111805	1	ITGA5	MF

toxic substance binding	1/51	11/16967	0.032581	0.149222	0.111805	1	CYP4B1	MF
vinculin binding	1/51	11/16967	0.032581	0.149222	0.111805	1	ACTN1	MF
UDP-glucosyltransferase activity	1/51	11/16967	0.032581	0.149222	0.111805	1	POGLUT2	MF
UDP-xylosyltransferase activity	1/51	11/16967	0.032581	0.149222	0.111805	1	POGLUT2	MF
xylosyltransferase activity	1/51	11/16967	0.032581	0.149222	0.111805	1	POGLUT2	MF
exopeptidase activity	2/51	97/16967	0.034403	0.150508	0.112769	2	GGTLC1/MMP14	MF
electron transfer activity	2/51	97/16967	0.034403	0.150508	0.112769	2	CYB5A/LOXL2	MF
omega peptidase activity	1/51	12/16967	0.035491	0.150508	0.112769	1	GGTLC1	MF
oligosaccharide binding	1/51	12/16967	0.035491	0.150508	0.112769	1	LOXL2	MF
oxidoreductase activity, acting on the CH-NH2 group of donors, oxygen as acceptor	1/51	13/16967	0.038392	0.158348	0.118642	1	LOXL2	MF
hydrolase activity	11/51	2052/16967	0.038723	0.158348	0.118642	11	ATP13A4/NAPSA/ATP8A1/GGTLC1/CHIA/PLA2G4F/SULF2/SULF1/ADAMTS12/MMP14/ADAM12	MF
heme binding	2/51	106/16967	0.040427	0.160241	0.120061	2	CYB5A/CYP4B1	MF
platelet-derived growth factor receptor binding	1/51	14/16967	0.041285	0.160241	0.120061	1	ITGA5	MF
metalloaminopeptidase activity	1/51	14/16967	0.041285	0.160241	0.120061	1	MMP14	MF
structural constituent of synapse	1/51	15/16967	0.044169	0.168578	0.126308	1	ACTN1	MF

lysophospholipase activity	1/51	16/16967	0.047045	0.168788	0.126465	1	PLA2G4F	MF
HMG box domain binding	1/51	16/16967	0.047045	0.168788	0.126465	1	PRRX1	MF
ion channel binding	2/51	116/16967	0.04755	0.168788	0.126465	2	GPD1L/ACTN1	MF
tetrapyrrole binding	2/51	116/16967	0.04755	0.168788	0.126465	2	CYB5A/CYP4B1	MF
oxidoreductase activity	5/51	662/16967	0.047909	0.168788	0.126465	5	SELENBP1/CYB5A/CYP4B1/ENOX1/LOXL2	MF
oxidoreductase activity, acting on the CH-NH2 group of donors	1/51	17/16967	0.049912	0.173178	0.129754	1	LOXL2	MF

Table S4: KEGG pathway functional enrichment of correlated genes.

Description	GeneRatio	BgRatio	P value	P adjust	q value	Count	Gene ID
Protein digestion and absorption	8/27	95/7914	5.89E-10	3.94E-08	3.28E-08	8	COL4A3/COL4A4/COL5A1/COL1A2/COL12A1/COL5A2/COL11A1/COL22A1

ECM-receptor interaction	5/27	88/7914	1.01E-05	0.000338	0.000282	5	COL4A3/COL4A4/THBS2/COL1A2/ITGA5
Focal adhesion	2/9	199/7914	4.46E-05	0.000997	0.00083	6	COL4A3/COL4A4/THBS2/COL1A2/ACTN1/ITGA5
Amoebiasis	4/27	102/7914	0.000363	0.006088	0.005069	4	COL4A3/COL4A4/COL1A2/ACTN1
AGE-RAGE signaling pathway in diabetic complications	1/9	100/7914	0.004595	0.051686	0.043038	3	COL4A3/COL4A4/COL1A2
Human papillomavirus infection	5/27	330/7914	0.004629	0.051686	0.043038	5	COL4A3/COL4A4/THBS2/COL1A2/ITGA5
PI3K-Akt signaling pathway	5/27	354/7914	0.006228	0.059609	0.049635	5	COL4A3/COL4A4/THBS2/COL1A2/ITGA5
Relaxin signaling pathway	1/9	129/7914	0.0093	0.07789	0.064857	3	COL4A3/COL4A4/COL1A2
Bile secretion	2/27	72/7914	0.024742	0.184192	0.153373	2	AQP4/SCTR
Proteoglycans in cancer	1/9	204/7914	0.031328	0.204311	0.170126	3	TWIST1/COL1A2/ITGA5
Sulfur metabolism	1/27	10/7914	0.033617	0.204311	0.170126	1	SELENBP1
Small cell lung cancer	2/27	92/7914	0.038871	0.204311	0.170126	2	COL4A3/COL4A4
GnRH signaling pathway	2/27	93/7914	0.039642	0.204311	0.170126	2	PLA2G4F/MMP14
Glycerophospholipid metabolism	2/27	97/7914	0.042789	0.204775	0.170512	2	GPD1L/PLA2G4F

Table S5: GSEA-KEGG analysis.

**Term**	**ES**	**NES**	**P value**	**FDR**	**FWER**
CELL_CYCLE	-0.6189	-1.9453	0.0021	0.1649	0.096
P53_SIGNALING_PATHWAY	-0.5126	-1.8166	0.004	0.2337	0.236

Table S6: GSEA- Hallmark analysis.

Term	ES	NES	P value	FDR	FWER
EPITHELIAL_MESENCHYMAL_TRANSITION	-0.7471	-1.9255	0.004	0.0737	0.072
UNFOLDED_PROTEIN_RESPONSE	-0.4192	-1.8135	0.052	0.0739	0.149
G2M_CHECKPOINT	-0.684	-1.8214	0.0124	0.0888	0.142
MITOTIC_SPINDLE	-0.5	-1.8433	0.0244	0.0959	0.122
GLYCOLYSIS	-0.446	-1.738	0.0059	0.1067	0.225
MTORC1_SIGNALING	-0.5102	-1.9514	0.01	0.1172	0.059
E2F_TARGETS	-0.6773	-1.6726	0.0192	0.1252	0.309
HYPOXIA	-0.4688	-1.6726	0.0211	0.1431	0.309
ANGIOGENESIS	-0.5743	-1.5631	0.0287	0.2205	0.473

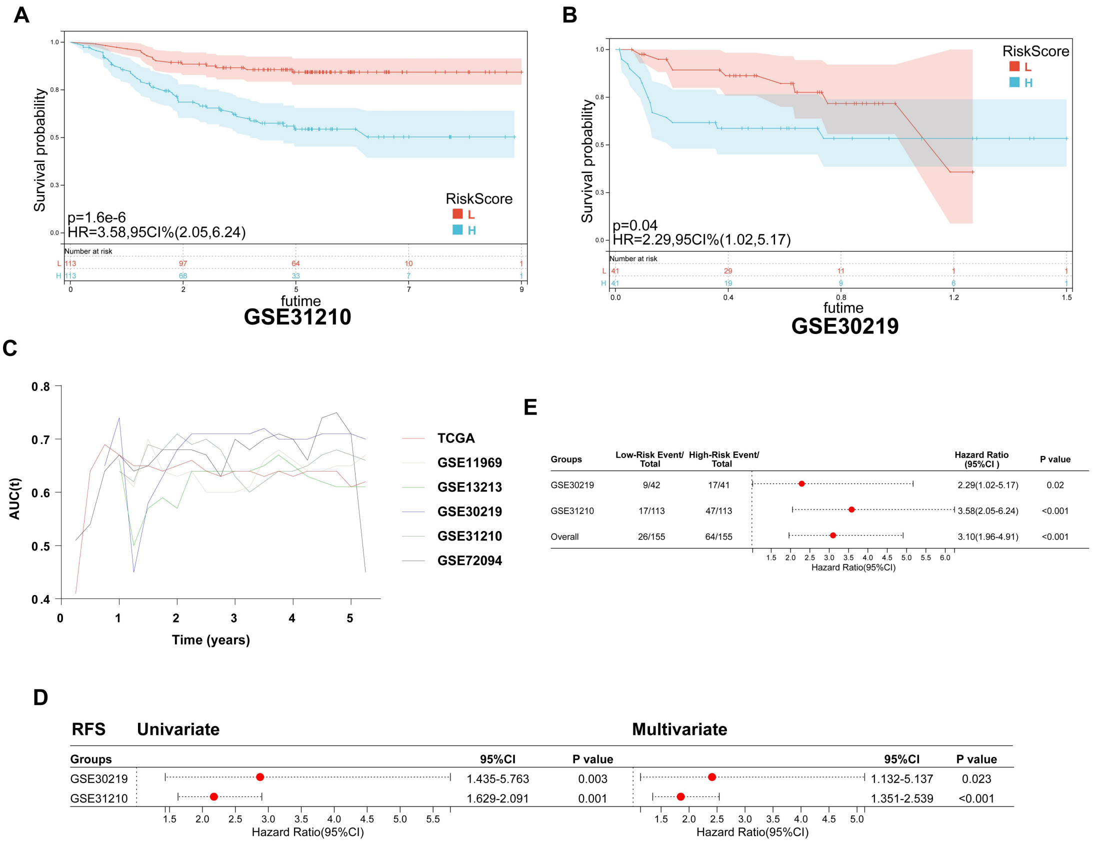

Figure S1: Survival analysis of Collagen-Risk model. **(A-B)** Validation of the prognostic value of Collagen-Risk model in two independent GEO datasets. **(C)** Time-dependent AUC value in TCGA, GSE11969, GSE13213, GSE30219, GSE31210, and GSE72094. **(D)** Forest plot of Cox analysis in 2 GEO datasets. For multivariate Cox regression analysis in GSE30219 dataset, HR value of Collagen-Risk model was adjusted by age, gender, and T stage. For multivariate Cox regression analysis in GSE31210 dataset, HR value of Collagen-Risk model was adjusted by age, gender, tumor stage, and smoking history. **(E)** A meta-analysis of verification result of two independent GEO datasets.

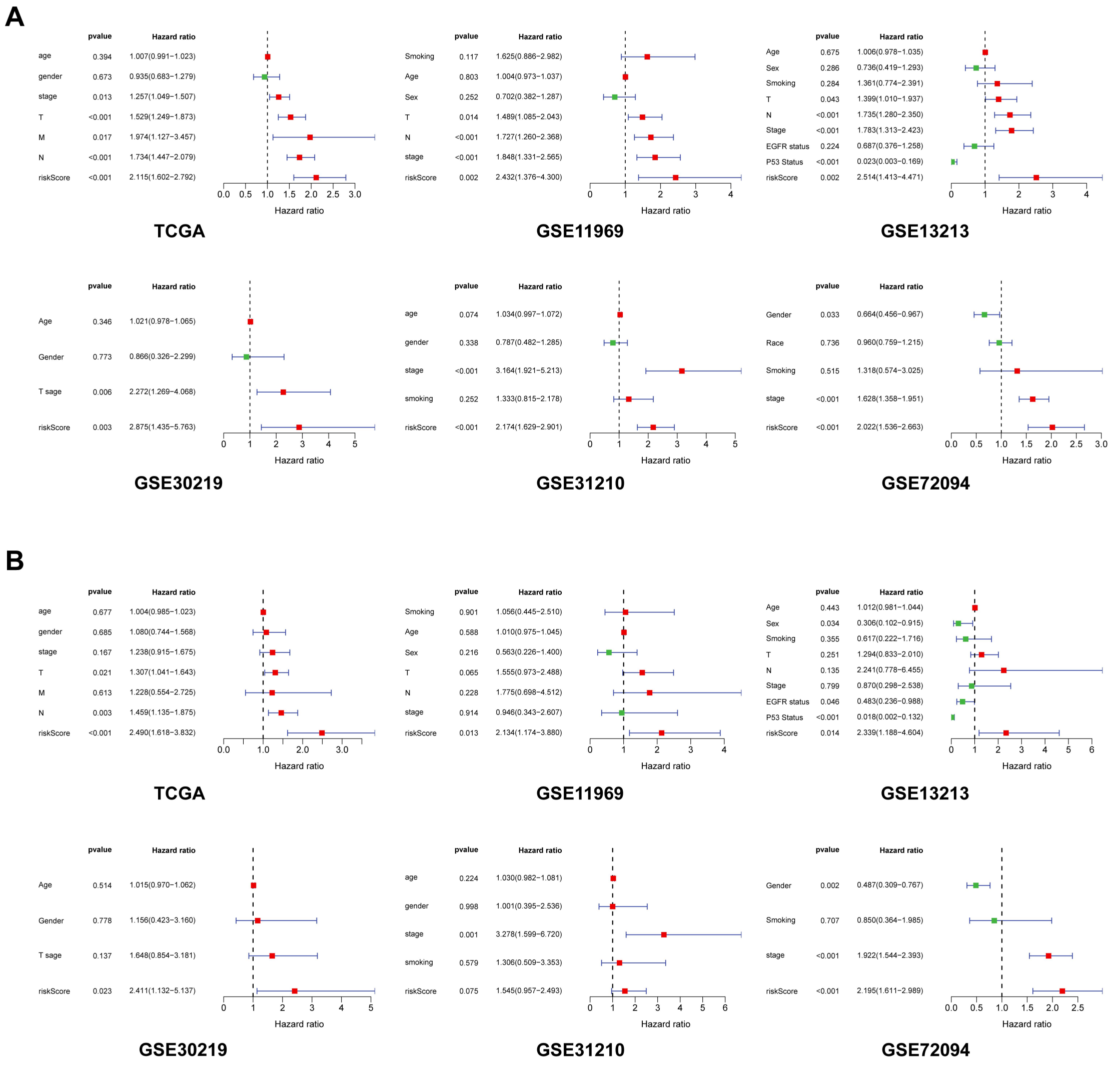

Figure S2: The prognostic value of clinical features in different datasets. **(A)** The results of univariate Cox analysis for TCGA and 5 GEO datasets. **(B)** The results of multivariate Cox analysis for TCGA and 5 GEO datasets.

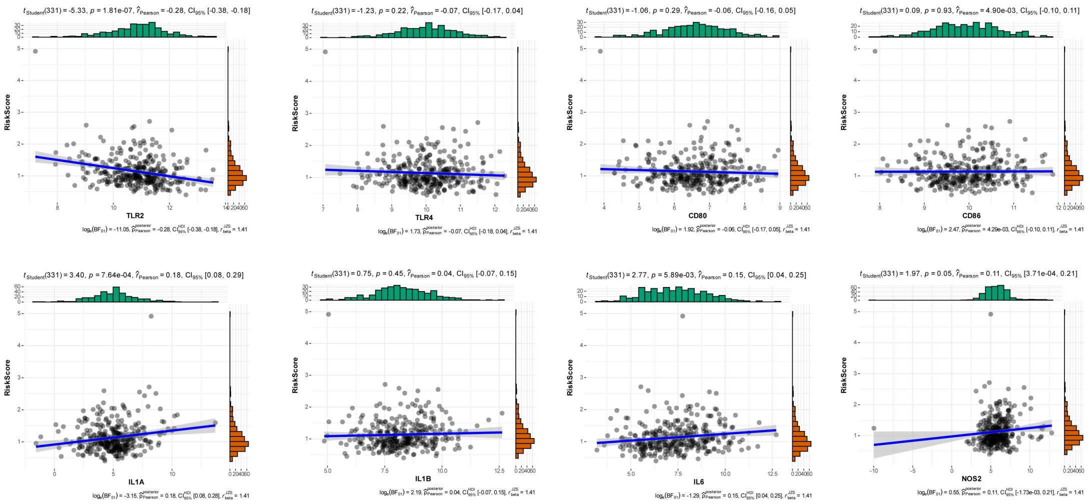

(A)

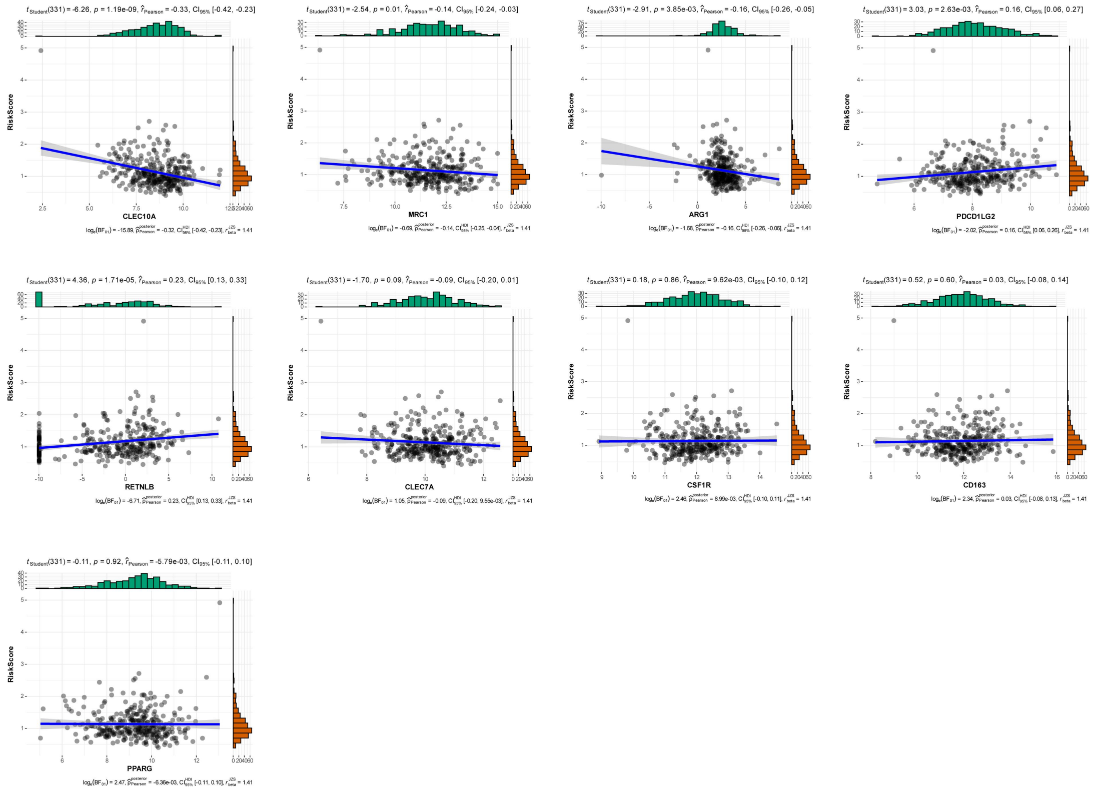

(B)

Figure S3: Collagen-Risk score was correlated with biomarker expression of M1 and M2 type macrophage. (**A**) M1 type macrophage: IL1a, IL1b, IL6, NOS2, TLR2, TLR4, CD80 and CD86. (**B**) M2 type macrophage: CSF1R, MRC1, PPARG, ARG1, CD163, CLEC10A, RETNLB, PDCD1LG2 and CLEC7A.

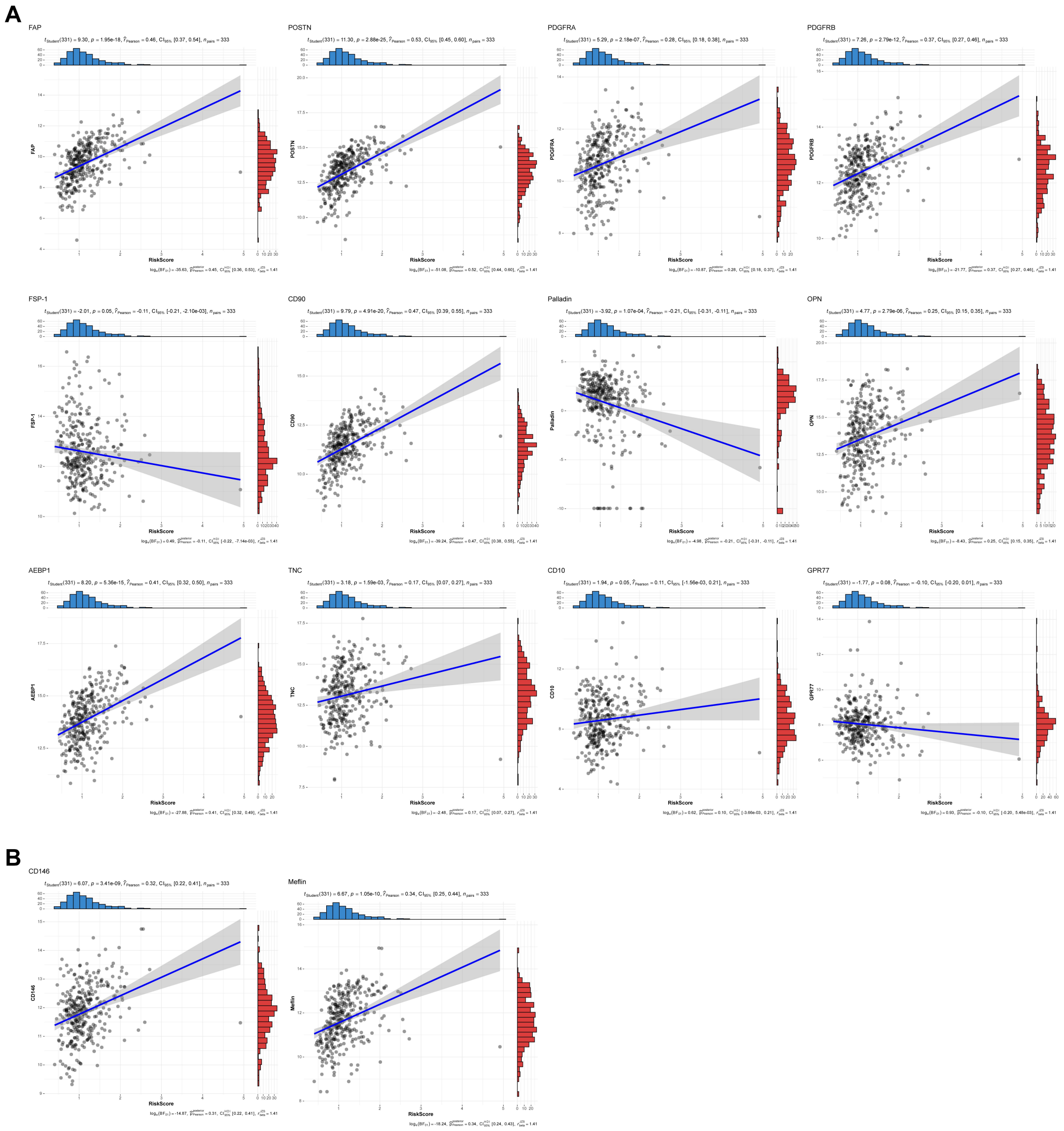

Figure S4: Collagen-Risk score was correlated with biomarker of CAFs. (**A**) Biomarker of cancer-promoting CAFs: FAP, POSTN, PDGFRα/β, FSP-1, CD90, Palladin, OPN, AEBP1, TNC, CD10 and GPR77. (**B**) Biomarker of cancer- restraining CAFs: Meflin and CD146.
